# A functional corona around extracellular vesicles enhances angiogenesis, skin regeneration and immunomodulation

**DOI:** 10.1002/jev2.12207

**Published:** 2022-04-09

**Authors:** Martin Wolf, Rodolphe W. Poupardin, Patricia Ebner‐Peking, André Cronemberger Andrade, Constantin Blöchl, Astrid Obermayer, Fausto Gueths Gomes, Balazs Vari, Nicole Maeding, Essi Eminger, Heide‐Marie Binder, Anna M. Raninger, Sarah Hochmann, Gabriele Brachtl, Andreas Spittler, Thomas Heuser, Racheli Ofir, Christian G. Huber, Zami Aberman, Katharina Schallmoser, Hans‐Dieter Volk, Dirk Strunk

**Affiliations:** ^1^ Cell Therapy Institute Spinal Cord Injury and Tissue Regeneration Centre Salzburg (SCI‐TReCS) Paracelsus Medical University (PMU) Salzburg Austria; ^2^ Department of Biosciences Paris Lodron University Salzburg Salzburg Austria; ^3^ Department of Transfusion Medicine and SCI‐TReCS PMU Salzburg Austria; ^4^ Core Facility Flow Cytometry and Department of Surgery Research Laboratories Medical University of Vienna Vienna Austria; ^5^ Vienna Biocentre Core Facilities Vienna Austria; ^6^ Pluristem Ltd. Haifa Israel; ^7^ Berlin Institute of Health at Charité – Universitätsmedizin BIH Centre for Regenerative Therapies (BCRT) Berlin Germany

**Keywords:** angiogenesis, EV corona, EV function, extracelular vesicle, placenta derived stromal cells, tangential flow filtration

## Abstract

Nanoparticles can acquire a plasma protein corona defining their biological identity. Corona functions were previously considered for cell‐derived extracellular vesicles (EVs). Here we demonstrate that nano‐sized EVs from therapy‐grade human placental‐expanded (PLX) stromal cells are surrounded by an imageable and functional protein corona when enriched with permissive technology. Scalable EV separation from cell‐secreted soluble factors via tangential flow‐filtration (TFF) and subtractive tandem mass‐tag (TMT) proteomics revealed significant enrichment of predominantly immunomodulatory and proangiogenic proteins. Western blot, calcein‐based flow cytometry, super‐resolution and electron microscopy verified EV identity. PLX‐EVs partly protected corona proteins from protease digestion. EVs significantly ameliorated human skin regeneration and angiogenesis in vivo, induced differential signalling in immune cells, and dose‐dependently inhibited T cell proliferation in vitro. Corona removal by size‐exclusion or ultracentrifugation abrogated angiogenesis. Re‐establishing an artificial corona by cloaking EVs with fluorescent albumin as a model protein or defined proangiogenic factors was depicted by super‐resolution microscopy, electron microscopy and zeta‐potential shift, and served as a proof‐of‐concept. Understanding EV corona formation will improve rational EV‐inspired nano‐therapy design.

## 1 INTRODUCTION

Extracellular vesicles (EVs) are a heterogeneous family of generally nanosized membrane‐coated vesicular structures derived from virtually all cell types (Riazifar et al., 2017). EVs comprise prototypic endosomal‐derived exosomes, assembled and released via multivesicular bodies, as well as outer cell membrane‐derived submicron sized ectosomes (microvesicles) and apoptotic bodies (Théry et al., 2018; Yáñez‐Mó et al., 2015). Conceptually, EVs transport their cargo to target sites enabling action over distance (Théry et al., 2018; Van Niel et al., 2018). Address codes on the EV surface may contribute to target specificity (Neri et al., 2018). In contrast, the biological identity of synthetic nanoparticles is critically defined by their corona acquired upon entry into protein‐rich environments. Albumin, representing the most abundant plasma protein, can act as a dysopsonin inhibiting nanoparticle uptake (Cedervall et al., 2007). Other distinct corona components can further decrease or increase cellular nanoparticle uptake in a biological environment (Ritz et al., 2015). It is meanwhile well established that rapid plasma protein corona formation, within seconds, can determine nanoparticle function (Tenzer et al., 2013). Vice versa, nanoparticles themselves can protect protein conjugates in their corona from protease degradation, thus also shaping functionality (Chan et al., 2019). This complex interplay impacts spatial and temporal distribution of nano‐therapeutics and may, at least in part, contribute to clinical failure of certain targeted nanomedicines (Witwer & Wolfram, 2021). The impact of a corona formation phenomenon on EV biology and function therefore needs to be addressed.

Peripheral artery disease (PAD) affects more than 15% of the > 80 year‐old population worldwide (Song et al., 2019). Critical limb ischemia (CLI) is an end‐stage of PAD resulting in high amputation rates and is associated with increased risk for cardiovascular events and death. Allogeneic placental‐expanded (PLX) stromal cells are currently evaluated in a clinical phase III trial (NCT03006770) for efficiency as an advanced CLI therapy (Norgren et al., 2019). The regenerative potential of placental cells is not restricted to CLI as evidenced by their hematopoietic support activity (Zahavi‐Goldstein et al., 2017), their capacity to protect from radiation injury (Pinzur et al., 2018), their current investigation for improving muscle regeneration in patients after hip arthroplasty (Winkler et al., 2018) and for treatment of coronavirus disease 2019 (COVID‐19) complications (clinical trial numbers: NCT04614025 and NCT04389450). Their mode of action was related to cytokine and growth factor secretion promoting angiogenesis, cell recruitment, migration and proliferation, resulting in tissue regeneration (Qazi et al., 2019). The immunomodulatory capacity of stromal cells has also been hypothesised to exert beneficial effects on local and systemic immune responses (Ankrum et al., 2014).

**FIGURE 1:**
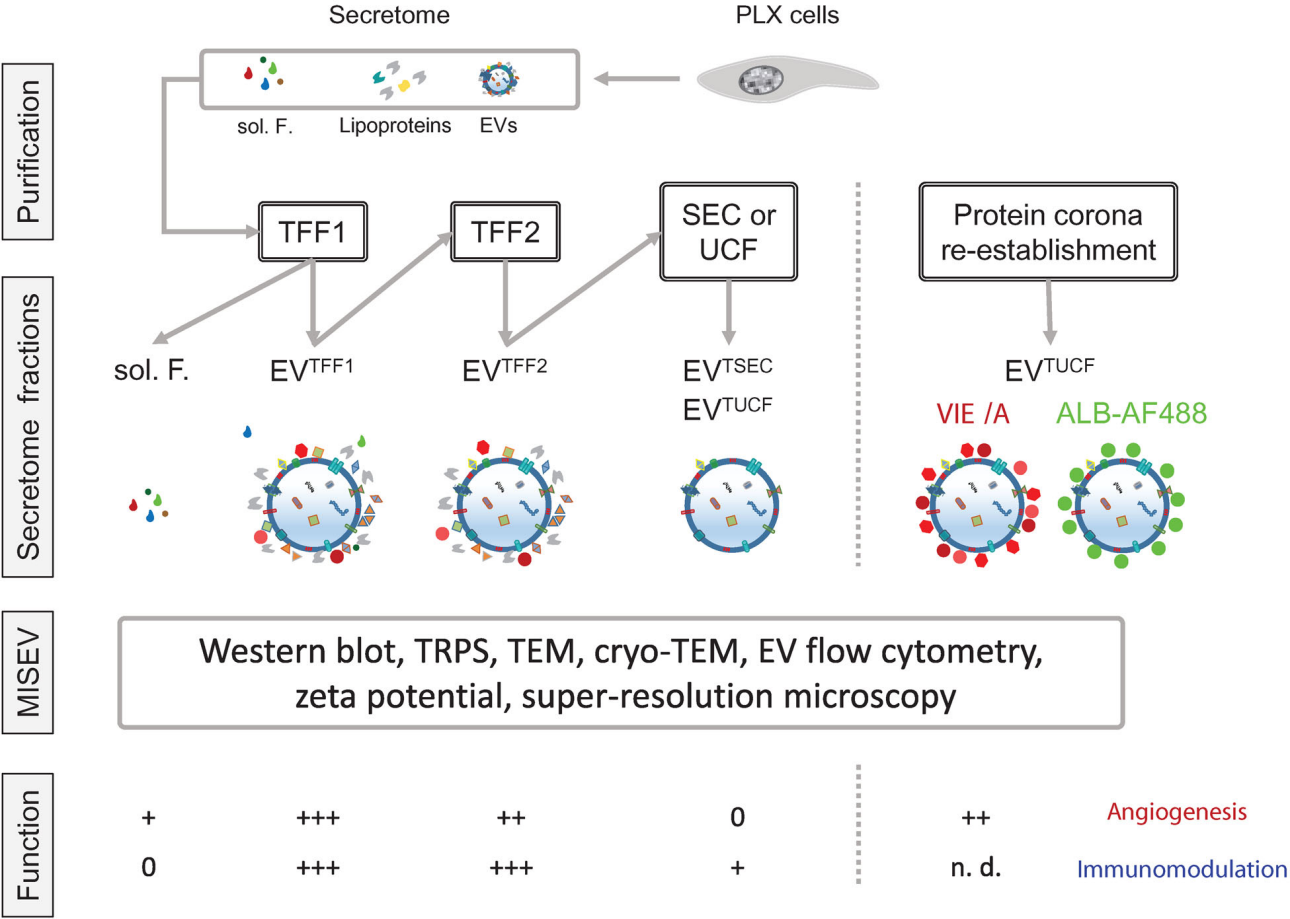
Experimental workflow. ALB, albumin; EVs, extracellular vesicles; MISEV, minimal information for studies of extracellular vesicles, updated 2018; SEC, size‐exclusion chromatography; sol. F., soluble factors; TEM, transmission electron microscopy; TFF, tangential flow filtration; TRPS, tunable resistive pulse sensing; TSEC, TFF x 2, followed by SEC; TUCF, TFF x 2 followed by ultracentrifugation (UCF); VIE/A, VEGF + IGF + EGF in albumin for corona reconstitution; EV characterization following MISEV2018 recommendations was performed as indicated in the results section and the respective figures. EV track ID: EV210462

This study was inspired by the multiplicity of EV functions during intercellular communication, paving their way toward clinical applicability, and partly replacing conventional cell‐based and nano‐therapies (Herrmann et al., 2021; Lener et al., 2015). We hypothesised that PLX‐EVs contribute to the mode of action of cell‐based therapy (Figure S1). We used conditioned media (CM) obtained after short‐term propagation of clinical grade PLX cell products (Norgren et al., 2019) under animal serum‐free particle‐depleted conditions to separate EVs from corresponding PLX‐derived soluble factors for comparative proteomic and functional analysis. Initial results confirmed proangiogenic and immunomodulatory potency of PLX‐EVs. Surprisingly, further EV purification by size exclusion chromatography (SEC) or ultracentrifugation (UCF) abrogated EV function, which could be re‐established by cloaking PLX‐EVs with a protein corona comprising three selected proangiogenic proteins dissolved in human albumin solution. An increased zeta‐potential indicated corona protein accumulation in close vicinity to the EVs and visualization by transmission electron microscopy (TEM) and super‐resolution microscopy confirmed corona formation. A schematic experimental workflow is shown in Figure 1.

## 2 MATERIALS AND METHODS

### 2.1 Ethics statement

Animal trial permission was given by local authorities according to Austrian legislation (§ 26 TVG 2012; animal trial number: BMBWF‐66.019/0032‐V/3b/2018). Human full‐thickness skin was obtained as biological waste material after informed consent as approved by the ethical committee of the region of Salzburg (vote number: 415‐E/1990/8‐216).

### 2.2 Cell culture media and reagents

Serum‐ or plasma‐containing media tested in this study included alpha‐modified minimum essential medium (α‐MEM), Eagle's MEM (EMEM, both Sigma‐Aldrich, USA) and endothelial growth medium (EGM2, Lonza, USA). Media were supplemented with 10% pooled human platelet lysate (HPL) or foetal bovine serum (FBS) as indicated, 5 mM N(2)‐L‐alanyl‐L‐glutamin (Dipeptiven, Fresenius Kabi, Austria) in the absence or presence of 2 U/ml preservative‐free heparin (Biochrom, Germany) without antibiotics or with 100 U/ml penicillin and 0.1 mg/ml streptomycin (both Sigma‐Aldrich, USA) as indicated in the results section. For endothelial cell culture, EGM‐2 was supplemented with hydrocortisone, fibroblast growth factor‐2 (FGF‐2), vascular endothelial growth factor (VEGF), insulin‐like growth factor 1 (IGF), epidermal growth factor (EGF) and ascorbic acid from the supplied bullet kit (all Lonza) and FBS was replaced by an equivalent volume of HPL (Burnouf et al., 2016). For particle depletion, media were prepared by clotting supplemented α‐MEM as described without heparin to avoid possible inhibition of EV function (Reinisch & Strunk, 2009). The collapsed fibrin clot was removed by centrifugation for 10 min, 3000 x g, at room temperature. The resulting precleared medium was filtered through a 0.22 μm stericup filter (Merck Millipore, USA) and finally particle depleted using a 1600 cm^2^ 500 kDa cut off hollow fibre modified polyethersulfone (mPES) membrane filter column operated on a KR2i tangential flow filtration (TFF) System (Repligen, USA). This medium was termed α‐MEM*/TFF. For comparison to TFF, we used UCF that was performed at 100,000 x g for 3 h in a Sorval WX80 ultracentrifuge with a T‐865 rotor (both Thermo Scientific, USA) as indicated.

Chemically defined and serum‐free media included CNT‐Prime (CELLnTEC, CH), X‐Vivo‐10, ‐15 and ‐20 (Lonza, USA) and MSC NutriStem XF medium (including a proprietary supplement mix; Biological Industries, Israel). Media were 0.22 μm sterile filtered prior to use.

### 2.3 PLX cell culture

Cryopreserved clinical grade PLX cell aliquots were obtained from Pluristem Ltd. (Israel) as part of a European funded research project (http://www.pace‐h2020.eu/). For this study, we used PLX cells derived from three individual donors (P150216R01, P250416R05 and P270114R27, termed P15, P25 and P27, respectively). For EV production, aliquots of 2.5 million PLX cells were propagated for one to two passages to avoid excess proliferation at moderate seeding density of 1000 cells/cm^2^ in 2528 cm^2^ cell factories (CF4, Thermo Fisher Scientific, USA) as established previously until approximately 70% confluence in α‐MEM/10% HPL (Bartmann et al., 2007; Schallmoser et al., 2007). For EV harvest, PLX cells were washed twice with 37°C prewarmed Dulbecco's phosphate‐buffered saline (PBS, Sigma Aldrich, USA) and cultured in particle‐depleted α‐MEM*/TFF for one to three additional 48 h periods with complete CM change.

### 2.4 EV quantification by tunable resistive pulse sensing (TRPS)

We used TRPS to quantify the particle content in various samples including fresh cell culture media, conditioned media, EV^TFF1^ and EV^TFF2^ preparations. Samples were diluted at least 1:1 in Dulbecco's PBS containing 0.05% Tween 20 that was also used as measurement electrolyte. Measurements were performed using a qNano Gold (Izon, New Zealand) equipped with an NP150 Nanopore (analysis size range 70–420 nm), operated at 47 ‐ 48 mm stretch and a pressure of 10 mbar. For ζ potential measurement, we used the same setup with a reduced pressure of 5 mbar. We used 200 nm polystyrene beads (Izon, New Zealand) as reference material and coated with 2 mg/ml albumin for corona formation (Fresenius Kabi, Austria).

### 2.5 EV enrichment by TFF and purification by SEC

To isolate and purify EVs from particle‐depleted CM, the conditioned medium was harvested after 48 h intervals. Possibly remaining cells were depleted by centrifugation at 300 x g for 5 min followed by a 3000 x g centrifugation step for 10 min to deplete cell debris. This precleared conditioned medium was first concentrated 100‐fold using a 300 kDa cut off hollow fibre mPES membrane filter column operated on a KR2i TFF System (Repligen, USA). The particle‐free soluble factor fraction was collected as permeate at this step while the TFF1 preparation was kept as ‘retentate’ inside the system. By washing this TFF1 fraction with twice the starting volume sodium chloride 0.9% buffered with 10 mM HEPES, an additional depletion of proteins and other nonvesicular nonparticulate content was obtained, resulting in more purified but still protein‐containing EV^TFF2^ fraction. SEC was performed using qEV 70 columns (Izon, New Zealand) according to manufacturer's instructions to further purify EVs and remove extravesicular putative soft corona protein (Figure S2). EVs were pooled from early fractions (7–9) whereas late fractions (17–19) were pooled as comparative corona‐derived protein samples.

### 2.6 EV corona removal by ultracentrifugation and re‐establishment of an EV corona

Ultracentrifugation is the standard method to deplete the corona around synthetic nanoparticles. EV^TFF2^ preparations were diluted 1:10 in sodium chloride 0.9% buffered with 10 mM HEPES and pelleted via ultracentrifugation at 4°C, 110,000 x g, for 90 min in a Sorval WX80 ultracentrifuge with a TH‐641 rotor at 25,400 rpm all (Thermo Fisher, USA). The resulting pellet was resuspended in the initial sample volume with sodium chloride 0.9% buffered with 10 mM HEPES. To re‐establish a protein corona on the ‘naked’ EV^TUCF^ they were incubated for 1 h at 37°C in EGM‐2 (Lonza) 4% of human serum albumin (Fresenius Kabi, Austria) and VEGF, IGF and EGF (termed VIE/A) (all from Lonza) at dilutions indicated in the figure legend. To re‐establish the protein corona on the naked EV^TUCF^ with fluorescently labelled proteins, BSA‐Alexa flour 488 (BSA‐AF488) (Thermo Fisher, USA), or VEGF165 (R&D Systems, USA) labelled with CF488A (Biotium,USA) according to manufactures protocol, EVs were incubated with 2 mg/ml BSA‐AF488 or 5 μg/ml VEGF‐CF488A for 1 h prior to antibody incubation for visualization of the corona via super resolution microscopy.

### 2.7 EV identity analysis by western blot and flow cytometry

To analyse EV identity and purity aspects, we performed western blotting using TGX stain free gradient 4–20% SDS‐PAGE gels run in a mini‐Protean system. Samples of 50 μg protein each were loaded with Laemmli buffer containing 50 μM dithiothreitol (DTT), as reducing agent, except for tetraspanins CD9, 63 and 81. After transfer using the Mini Trans‐Blot tank system (all Bio‐Rad, USA), nitrocellulose membranes were probed with primary antibodies diluted in Tris buffered saline with Tween 20 detergent (TBST) including 2% bovine serum albumin (BSA) in the dilutions indicated in Table S1.

Detection was performed using horseradish peroxidase (HRP)‐labelled secondary antibodies (rabbit antimouse IgG, A27025, Thermo Fisher, USA; mouse antigoat 205‐035‐108, Jackson Laboratories, USA; or polymer goat‐antirabbit, K4002, DAKO EnVision, Agilent, USA) depending on the host species of the primary antibody and clarity enhanced chemiluminescence (ECL) substrate. Bands were visualised and quantified using a ChemiDoc system and image lab software (all Bio‐Rad). Densitometry of specific bands was quantified after background correction in relation to the total protein content detected with the stain free technology before transfer.

In order to obtain a broader overview of markers present on the surface of PLX‐derived EVs we applied a bead‐based screening assay measured with flow cytometry as described (Koliha et al., 2016). To standardise the EV input for the assay, we loaded 1 × 10^9^ EV^TFF1/TFF2^ on the MACS Plex capture bead mix (Miltenyi, Germany) (Wiklander et al., 2018) stained with a mix of CD9‐, CD63‐ and CD81‐ allophycocyanin (APC) detection reagent mix according to manufacturer's protocol. Analysis was performed with a LSR Fortessa instrument (BD, USA) equipped with 355 nm, 405 nm, 488 nm, 561 nm and 640 nm lasers. Raw measurement data were corrected for unspecific binding of the detection antibody mix to beads and expressed as relative fold‐change of mean fluorescence intensity (MFI) compared to samples stained with isotype control (Figure S3A).

For single EV analysis by flow cytometry EV^TFF1^ and EV^TFF2^ preparations were stained with lactadherin‐Alexa Fluor 647 (CellSystems, Germany), and calcein AM (Sigma‐Aldrich, USA) that were aggregate‐depleted by centrifugation at 17,000 x g for 10 min directly prior to use. Staining with lactadherin was performed for 30 min at 4°C and subsequently, after 1:10 dilution of the samples, with calcein for 5 min at room temperature. Samples were further diluted 1:5 in PBS and analysed on a Cytoflex flow cytometer (Beckman Coulter, USA). For creating a size‐based gate < 1000 nm for EV detection, we used 100, 200, 500 and 1000 nm green fluorescent‐labelled silica beads (Kisker Biotech, Germany) as illustrated in Figure S3B. Based on the separation of fluorescent EVs from particle background we only recorded fluorescence‐positive events using double‐fluorescence triggering in the fluorescein isothiocyanate (FITC) and APC channels. For quantifying calcein^+^ and lactadherin^+^ EVs, gating was performed using staining reagents appropriately diluted in PBS as a negative control. Percentage of calcein^+^, lactadherin^+^ and double‐positive populations was calculated as a part of the sum of all acquired events and were displayed as a pie chart (Figure S3C).

### 2.8 Electron microscopy

For conventional negative contrast TEM, 10 μl EV samples were applied on formvar‐coated 100 mesh copper grids (Agar scientific, UK), fixed with 2.5% glutaraldehyde and stained with uranyl acetate replacement solution 1:10 (Electron Microscopic Sciences, UK) in bidistilled water. Dried samples were imaged using an 80 kV LEO EM 910 transmission electron microscope (Zeiss, Germany) equipped with a Tröndle 227 Sharp Eye digital camera system. To quantify EV size and EV corona thickness based on these negative contrast TEM images, we used ImageJ software. For each EV the perimeter (P) of the dense EV „core and the whole EV including the corona contrast was determined. The radius r for both parameters was determined (r = P/(2π)) and the thickness of the EV corona was calculated as the difference between the whole EV and the EV core (corona thickness = r(whole EV)‐r(EV core)). Between 36 (EV^TFF2^) and 56 (EV^TUCF_VIE/A^) EVs from images obtained from EVs from three independent PLX donors were analysed for corona thickness determination.

For cryo‐TEM, EV samples were diluted 1:10 in 0.9% sodium chloride solution and 4 μl were applied to Quantifoil (Großlöbichau, Germany) Cu 400 mesh R1.2/1.3 holy carbon grids (Leica Microsystems, Germany). Grids were glow discharged for 1 min at ‐25 mA with a Bal‐Tec (Balzers, Liechtenstein) SCD005 glow discharger and loaded into a Leica GP grid plunger with the climate chamber set at 4°C and 70% relative humidity. EV samples were diluted 1:10 in 0.9% sodium chloride solution and 4 μl were applied to the carbon side of the grid. After front‐side blotting for 2–8 s (using the instrument's sensor function, no pre‐ or postblotting incubation) with Whatman filter paper #1 (Little Chalfont, Great Britain) grids were plunge frozen into liquid ethane at approximately 180°C for instant vitrification. Cryo‐samples were transferred to a Glacios cryo‐transmission microscope (Thermo Scientific, USA) equipped with an X‐FEG and a Falcon III direct electron detector (4096 × 4096 pixels). The microscope was operated in a low‐dose mode using the SerialEM software (Mastronarde, 2005). Images were recorded digitally in linear mode of the Falcon III camera at magnifications of 5300 (pixel size: 27.5 Å, defocus: ‐50 μm, dose: 0.2 e/Å2), 36,000 (pixel size: 4.1 Å, defocus: ‐6 μm, dose: 14 e/Å2) and 150,000 (pixel size: 0.98 Å, defocus: ‐3 μm, dose: 60 e/Å2).

### 2.9 Super‐resolution microscopy

For super‐resolution analysis of EVs, samples were immobilised on microfluidic glass slides (EV profiler Kit, Oxford Nanoimaging, UK) and stained with CD9‐ATTO488, CD63‐Cy3 and CD81‐AlexaFluor 647 according to manufacturer's instructions. Images were acquired via direct stochastic optical reconstruction microscopy (dSTORM; Nanoimager S, Oxford Nanoimaging, UK) using 30%, 40% and 50% power on the 488 nm, 561 nm and 640 nm laser, respectively. Per channel 2500 images were recorded for localization mapping. Co‐localizations of the different tetraspanins were analysed using the CODI platform (https://alto.codi.bio/). Localization clusters showing more than 10 individual localizations were considered as EVs. EVs were considered positive for a marker when more than 10 individual localizations were detected in the same channel in a radius of 60 nm around the centre of a cluster. For statistical analysis, three fields of view were acquired for three independent donors. To visualise the protein corona around EVs, EV^TUCF^ coated with a fluorescent albumin‐AF488 or VEGF‐CF488A corona were stained with tetraspanin CD9, (R&D Systems, USA) CD63, (Becton Dickinson, USA) and CD81 (R&D Systems, USA), all conjugated to Alexa flour 647 (tetramix) as published (Gomes et al., 2022). Quantification of colocalizations was performed as reported with a limit of 150 nm radius and five localizations for quantifying an event as colocalization.

### 2.10 Tandem mass tag (TMT) proteomics and bioinformatics

Reagents included acetonitrile (≥ 99.9%) and methanol (≥ 99.9%; both VWR, Austria), 1,2‐dithiothreitol (DTT; ≥ 99.5%), formic acid (FA; 98 ‐ 100%), iodoacetamide (IAA; ≥ 99.0%), sodium dodecyl sulphate (SDS; ≥ 99.5%), triethyl ammonium bicarbonate (TEAB, 1 mol/L) and trifluoroacetic acid (≥ 99.0%; all Sigma‐Aldrich, Austria), ortho‐phosphoric acid (85%; Merck, USA), and sequencing grade modified trypsin (Promega, USA). Deionised water was purified with a MilliQ Integral 3 instrument (Millipore, USA).

To determine protein content, samples were adjusted to 5% SDS and 50 mmol/L TEAB (pH 7.55) and incubated at 95°C for 10 min to lyse the EVs. Samples were analysed by a Pierce bicinchoninic acid protein assay kit (Thermo Fisher Scientific, Austria) according to the manufacturer´s instructions. S‐Trap mini columns (Protifi, Huntington, NY, USA) were utilised for sample preparation and 100 μg of protein were prepared according to the manufacturer´s instructions with minor adjustments: Lysis of EVs as well as denaturation and reduction of proteins were performed in 5% SDS and 50 mmol/L TEAB supplemented with 40 mM DTT at 95°C for 10 min. Cysteines were alkylated by the addition of IAA to a final concentration of 80 mM and incubated in the dark for 30 min. Proteins were digested within the S‐Trap matrix with trypsin at an enzyme to substrate ratio of 1:10 w/w at 37°C for 18 h. Peptides were eluted and subsequently dried using a vacuum centrifuge. These samples were resuspended in H_2_O + 0.1% FA to a concentration of 3.33 mg/ml. Peptides of each sample (20 μg) were labelled by a TMT 10‐plex kit (Thermo Fisher Scientific, Austria). Labelled samples were pooled and desalted using 100 μl Pierce C18 tips (Thermo Fisher Scientific, Austria) and dried again using a vacuum centrifuge. These samples were resuspended in H_2_O + 0.1% FA to a concentration of 5 mg/ml.

High‐performance liquid chromatography (HPLC) separation was carried out on a nanoHPLC instrument (UltiMate U3000 RSLCnano, Thermo Scientific, Germany) at a flow rate of 300 nl/min and a column oven temperature of 50°C. Separation of unlabelled samples was performed on an Acclaim PepMap 100 C18 column (500 mm x 75 μm i.d., 3 μm particle size, Thermo Fisher Scientific, Austria). Samples (3.3 mg/ml; 0.15 μl) were injected using a microliter pick‐up mode (loop volume 1 μl). A multistep linear gradient of mobile phase solutions A (H_2_O + 0.1% FA) and B (acetonitrile + 0.1% formic acid, FA) was applied as follows: 1–22% B for 200 min, 22–30% B for 40 min, 30–55% for 30 min, 90% B for 20 min and 1% B for 40 min. Each sample was measured once.

Separation of TMT‐labelled samples was performed on a 2000 mm μPAC C18 column (PharmaFluidics, Ghent, Belgium). The sample [5.0 mg/ml; 1 μl] was injected using a microliter pick‐up mode (5 μl loop volume). A multistep linear gradient of mobile phase solutions A and B were applied as follows: 1–22% B for 500 min, 22–40% B for 100 min, 90% B for 30 min and 1% B for 100 min. Five technical replicates were measured.

All mass spectrometry measurements were conducted in positive ion mode on a hybrid mass spectrometer (QExactive Plus benchtop quadrupole‐Orbitrap mass spectrometer) equipped with a Nanospray Flex ion source (both Thermo Scientific, Germany) and a SilicaTip emitter with 360 μm outer diameter, 20 μm inner diameter, and a 10 μm inner tip diameter (New Objective, Woburn, MA, USA). Mass spectrometric data were acquired with the following instrument settings: spray voltage of 1.5 kV, capillary temperature of 320°C, S‐lens, radio frequency level 55.0, MS1 AGC target 3 × 10^6^, m/z range 400–2000, maximum injection time of 100 ms, resolution of 70,000 at 200 m/z. Data‐dependent tandem mass spectrometry was carried out in the higher‐energy collisional dissociation (HCD) cell at a normalised collision energy (NCE) setting of 28.0 and a resolution setting of 17,500 at m/z 200 for unlabelled samples and at a resolution of 35,000 at m/z 200 for TMT‐labelled samples. The top 15 signals were chosen for fragmentation with a 2.0 m/z isolation window, an automatic gain control and maximum injection time of 100 msec. The dynamic exclusion was set to 30 s. The instrument was calibrated using Pierce LTQ Velos ESI Positive Ion Calibration Solution (Life Technologies, Vienna, Austria).

All data were evaluated using MaxQuant software (version 1.6.1.0) using default settings. A protein list was obtained from the Uniprot database including both Swiss–Prot as well as TrEMBL entries for homo sapiens (access: 10.03.2019) and was provided for MaxQuant searches (Cox & Mann, 2008; The UniProt Consortium 2017). TMT‐labelled data were further processed using Perseus software package (version 1.6.1). Only protein groups with 10 quantified channels were included for analysis, log2‐transformed and normalised by subtraction of the median. Analysis of the TMT‐labelled samples was conducted using Ingenuity Pathway Analysis (IPA; version 47547484; Qiagen Bioinformatics, Redwood City. CA, USA).

R software (www.R‐project.org) was used all further proteomics analysis. For the TMTP data, contaminants were removed and values were log2‐transformed and normalised by subtraction of the median of each channel. In order to see how samples cluster together, a principle component analyses (PCA) and hierarchical clustering analysis using Euclidean distance were conducted on the whole normalised dataset. Differential expression analysis was conducted using limma package and p‐values were corrected using Benjamini and Hochberg multiple testing correction. Proteins were considered significantly differentially expressed if the corrected Benjamini and Hochberg p‐value was < 0.05 and absolute log2 fold change > 0.6. Data from the label‐free proteomics analysis were used in order to detect proteins present in specific fractions. Samples were considered detected in a specific fraction if they were present in all three replicates. In order to estimate the biological process gene ontology (GO) terms enriched in the purified EV fraction, compared to the soluble factors, we combined the proteins that were significantly enriched in the EV fraction compared to the soluble factors (in the TMTP analysis) and/or found only in the EV fraction using the label‐free proteomics analysis. Enrichment analysis was conducted using ClusterProfiler R package and GO were considered significantly enriched if the adjusted p‐value was < 0.01 and the gene count was > 5. Only the most significantly enriched proteins were shown (fold enrichment > 4).

### 2.11 Angiogenesis and immunomodulation

To assay angiogenic potential of different PLX secretome fractions we used a vascular–like network formation assay on matrigel as previously described (Reinisch et al., 2009). Umbilical cord blood (UCB)‐derived endothelial colony forming cells (ECFCs) were seeded on top of matrigel (angiogenesis assay kit, Merck Millipore, USA; in Figure 4a) or a reduced growth factor basement membrane matrix (Geltrex, Thermo Fisher, USA; in Figure 4b) in an angiogenesis 96 well μ‐plate (Ibidi, Germany) at a density of 31,500 cells/cm^2^. Cells were treated with EV preparations in an EV to ECFC ratio of 10,000:1, 1000:1, 100:1 and 10:1, or with the volume equivalent to EV‐free PLX stromal cell‐derived soluble factors. Completely supplemented EGM‐2 (Lonza) served as positive control and nonsupplemented EBM‐2 with 2 or 4% of human serum albumin (Fresenius Kabi, Austria) as negative control. For investigating proteolytic stability of EV preparations vs. sol. F. both were treated at equal protein amounts with 0.25% Trypsin (Gibco, Thermo Fisher, USA) for 30 min, followed by inhibition of trypsin with 1 mM phenylmethylsulfonyl fluoride serine protease inhibitor (Thermo Fisher, USA), overnight, before applying on ECFCs to monitor network formation capacity on matrigel. To evaluate the importance of VEGF for EV's angiogenic potential, VEGF was blocked with anti‐VEGF antibody (Avastin; Roche, Switzerland)) at 100 μg/ml for 1 h before sample application. Images were taken every hour for 12 h on an Eclipse Ti inverted microscope (Nikon) equipped with a custom‐build live cell incubation system (Oko Lab, Italy and Nikon, Austria) using a 4x objective. Images were processed with the NIS Elements Advanced Research package analysis software (Nikon). Total matrigel areas were cut out of raw images, homogenised (strength 16), and subjected to intensity equalization. Afterwards, pictures were sharpened slightly and denoised (advanced denoising 5.0). Finally, lookup tables were adjusted to 3000–13,000 and images were exported as TIFF files. Exported images were cut at the diameter of 1300 pixels to remove edges of the plate and the contrast was enhanced. Processed pictures were analysed to automatically detect the network structures with Image J using the Angiogenesis Analyzer plugin and total length of tube‐like structures was determined (https://imagej.nih.gov/ij/macros/toolsets/Angiogenesis%20Analyzer.txt).

The effect of PLX‐derived EVs on the immune response was analysed using a T cell proliferation assay as published (Ketterl et al., 2015; Pachler et al., 2017). In brief, peripheral blood mononuclear cells (PBMCs) were isolated and pooled from 10 individual donors before labelling with carboxyfluorescein succinimidylester (CSFE; Sigma‐ Aldrich, USA) and cryopreservation in appropriate aliquots for later use. These prelabelled PBMCs (300,000 per flat‐bottomed 96‐well plate) were stimulated with 5 μg/ml phytohemagglutinin (PHA, Sigma‐Aldrich, USA) to induce mitogenesis (at day four). Assays were once loaded with EV doses at three‐fold serial dilution in a ratio of 15,000:1, 5000:1, 1666, or 555:1, or corresponding volumes of particle free soluble factors. In case parental cells were used for inhibition of T cell proliferation, PLX cell to PBMC target cell ratios were 1:1, 1:3, 1:9 and 1:27, respectively. The percentage of proliferating T cells was measured by flow cytometry as the fraction of viable CD3 positive cells with reduced CFSE staining compared to non PHA‐stimulated cells. Inhibition of T cell proliferation was expressed as percentage relative to maximum proliferation without EV addition.

### 2.12 Proteolytic stability and VEGF immunoassay

To investigate the proteolytic stability of proangiogenic and immunomodulatory factors in EV preparations compared to soluble factors, samples were mixed 1:1 with cell culture grade 0.25% trypsin solution (Gibco, Thermo Fisher, USA) and incubated as indicated. To block remaining trypsin, activity reaction was stopped by adding Halt protease inhibitor cocktail or 1 mM phenylmethylsulfonyl fluoride serine protease inhibitor (Thermo Fisher, USA). VEGF concentration in EV^TFF2^ vs. EV^TUCF^ was measured in duplicates for each sample (*n* = 3) according to manufacturer's instructions (Abcam; ab100663 – VEGF human ELISA kit) using a Spark microplate reader /Tecan, Austria) at OD 450 nm. To determine stability of different proangiogenic factors in samples before and after protease treatment 300 μg of total protein was loaded on each membrane of a sandwich western blot array (Proteome Profiler Human Angiogenesis Kit, ARY007, R&D Systems, USA) and processed according to manufacturer's instructions. For quantification, membranes were scanned with a ChemiDoc system (Bio‐Rad, USA) and [Area x Intensity] of individual spots was measured using ImageLab software (Bio‐Rad, USA)

### 2.13 EV uptake and signalling in immune cells

To test the uptake of EVs into PBMCs by flow cytometry and confocal microscopy and to question a hypothetic cell tropism, EVs were labelled with BODIPY FL C5‐ceramide complexed to BSA (5 μg/ml; Thermo Fisher, USA) for 1 h at 4°C. To remove unbound dye, samples were diluted 1:10 with 0.9% sodium chloride solution containing 10 mM HEPES and centrifuged at 4°C with 110,000 x g for 90 min in a Sorval WX80 ultracentrifuge with a TH‐641 rotor at 25,400 rpm all (Thermo Fisher, USA). The pellet was resuspended in the initial sample volume with 0.9% sodium chloride solution containing 10 mM HEPES. As a control for dye aggregates in the uptake experiment, dye in buffer alone was processed accordingly as a negative control sample. Particle concentration was determined thereafter by TRPS to adjust EV uptake number. PBMCs were cultured overnight in RPMI supplemented with 10% human AB‐serum, 5 mM dipeptiven, 10 nM HEPES and 100 U/ml penicillin and 0.1 mg/ml streptomycin, in an Erlenmeyer flask at a density of 1 × 10^6^ cells/ml. After washing in PBS, unspecific binding was blocked using sheep serum and cells were stained with CD3‐eF450 (2 μg/ml), CD19‐APC (0.25 μg/ml), CD56‐PE‐Cy7 (0.5 μg/ml; all eBioscience, USA), CD14‐APC‐H7 (0.5 μg/ml; BD) and 7‐AAD (1:100; eBioscience). Washed cells were resuspended in PBS containing 5% sheep serum and were sorted accordingly. Sort‐purified cells were incubated with stained EVs for 24 h, fixed with 4% formalin and investigated for uptake with a laser scanning confocal microscope (Axio Observer Z1 attached to LSM700, Carl Zeiss).

To quantify the percentage and intensity of EV uptake, 300,000 PBMCs were incubated for 1 h, 24 h or 48 h with stained EVs or dye control sample in a ratio of EV to PBMC of 5000:1. For flow cytometric quantification of EV uptake, PBMCs were washed with PBS, blocked with 2% sheep serum and stained with antibodies as indicated in the Table S2. Cells were analysed with a 3‐laser 10‐color Gallios Flow cytometer (Beckman Coulter, USA).

Cell‐type specific response to EV or soluble factor stimulation was investigated with sorted T cells and monocytes. Incubating equal sorted cell numbers for 15 min in a 10,000:1 ratio with TFF2 EVs or the corresponding volume of soluble factors was followed by cell lysis and analysis of the cell's phospho‐kinome with the Human Phospho Kinase array Kit, ARY003C, (Human Phospho‐RTK, R&D Systems, USA) according to manufacturer's protocol.

### 2.14 Skin grafting

Immune‐deficient NOD.Cg‐Prkdcscid Il2rgtm1WjI/SzJ mice (614NSG, Charles River) were used. Single‐cell suspension transplants containing 6 × 10^6^ keratinocytes and 6 × 10^6^ fibroblasts diluted in 200 μl α‐MEM/10% FBS (total volume 400 μl) were transplanted onto a full thickness back skin wound. Keratinocyte and fibroblast isolation and cultivation, and the grafting procedure were performed as previously described (Ebner‐Peking et al., 2021). In order to evaluate EV–induced angiogenesis, 200 μl of TFF2‐EVs or equivalent volume of soluble factors from PLX cells obtained after separating TFF1‐EVs, cultivated in α‐MEM/10% particle‐depleted FBS, were added to the cell mixture before grafting. Skin biopsies were taken 14 days after initial grafting, fixed in 4% formaldehyde and prepared for histology.

### 2.15 Histology of skin sections, image acquisition and quantification

For histochemistry and immune‐histochemistry, paraffin‐embedded skin samples were cut into 4 μm sections. Haematoxylin and eosin (HE) staining using Mayer's Hemalaun (1.09249.2500, Merck) and Eosin Y (1.15935.0100, Merck) was done in a linear slide stainer (Leica ST4040). For Masson–Goldner trichrome staining, kits were used according to the manufacturer's recommendations (12043, 14604, Morhisto). Automatic scanning of full slides in 40x magnification was done using the VS‐120‐L Olympus slide scanner 100‐W system and processed using the Olympus VS‐ASW‐L100 program. Evaluation of the epidermal thickness and murine vessel quantification was done according to published work (Ebner‐Peking et al., 2021), both measured in the Olympus VS‐ASM‐L100 program. Per group, four biological and at least three technical replicates were included.

### 2.16 Statistics

Statistical analysis of the results was performed using One‐Way ANOVA analysis of variance with a confidence interval of 95% and corrected for multiple comparisons using the Holm Sidak algorithm in GraphPad Prism version 7.03. Proteomic results were analysed using R. P values < 0.05 were defined as significant.

## 3 RESULTS

### 3.1 Physically defined media with a low particle count permit cell‐derived EV analysis

In initial experiments, we determined the particle content of standard media for stromal cell culture, because high pre‐existing particle concentrations would disable PLX‐EV purification. In fact, α‐MEM and other conventional media supplemented with either FBS or pooled HPL (Schallmoser & Strunk, 2009) contained mean 4 × 10^8^–3 × 10^9^ particles/ml. Fibrinogen‐depleted α‐MEM (α‐MEM*), which could be used without heparin that otherwise might inhibit EV uptake (Colombo et al., 2014; Laner‐Plamberger et al., 2015), had even more particles of up to 10^10^/ml. Both UCF and TFF allowed for significant depletion of the mostly cell culture supplement‐derived EVs. We chose TFF in further experiments for better scalability and time saving purposes because efficient depletion of serum‐EVs by UCF required up to 24 h centrifugation at 100,000 x g and was restricted to limited volume (Shelke et al., 2014). Some but not all tested chemically defined serum‐free media contained less than 10^8^ particles/ml (Figure S4A). Culturing PLX cells in fibrinogen‐containing α‐MEM/HPL resulted in a significant rise of particles measured on top of the pre‐existing mostly HPL‐derived EVs within 6 days. We cannot exclude that fibrin aggregates, as shown recently (Tóth et al., 2021), contribute to this higher particle count. PLX cell culture in TFF particle‐depleted α‐MEM*/TFF or in defined media resulted in significantly elevated particle counts after 6 days indicating effective release of PLX‐EVs under these conditions (Figure S4B). TRPS analysis showed a more heterogeneous particle size distribution under serum‐free conditions including measurably larger particles (Figure S4CD). For reasons of efficiency, we chose fibrinogen‐depleted and particle‐depleted α‐MEM*/TFF for subsequent experiments.

### 3.2 Scalable TFF strategy for enriching cell‐derived functional EVs

We next devised a scalable EV production process (Figure S3) based on previous results (Pachler et al., 2017). Fibrinogen‐depleted α‐MEM* (n x 500 ml) was depleted for pre‐existing particles (including EVs) before use in large‐scale PLX short‐term culture. CM was harvested from cell factories after 48 h periods in particle‐depleted α‐MEM*/TFF and subjected to 100x concentration for PLX‐EV enrichment (termed EV^TFF1^) and simultaneous soluble factor separation. Isovolumetric washing of EV^TFF1^ removed remaining soluble factors (creating EV^TFF2^).

**FIGURE 2:**
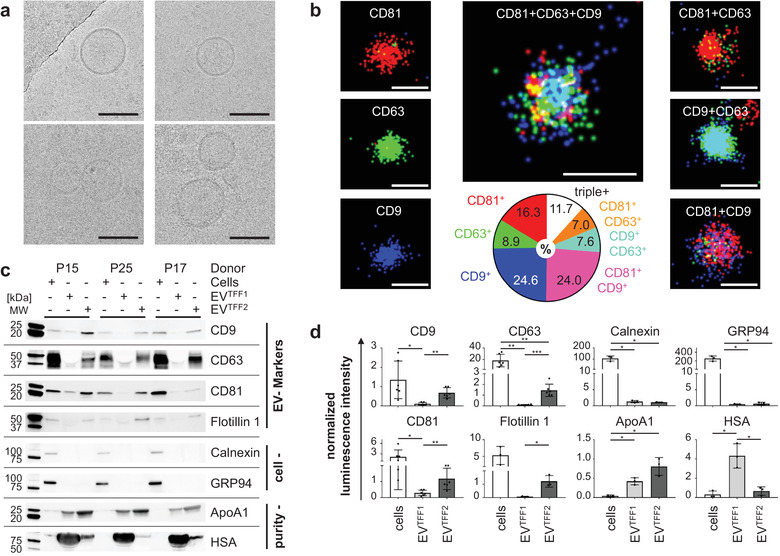
EV identity and purity. (a) Cryo‐TEM showing representative images of PLX‐EVs after TFF2 (EV^TFF2^). Scale bars 100 nm. (b) Super‐resolution microscopy topology display of tetraspanins CD81 (red), CD63 (green) and CD9 (blue), via single‐molecule fluorescence of individual EV^TFF2^ (left) and the overall distribution of double and triple marker positive EVs as indicated in text inserts, based on 99,209 EV areas analysed. Scale bars 100 nm. (c) Western blots comparing placental‐expanded (PLX) cells (from lots P15, P25, P27) and corresponding EV preparations after one or two TFF cycles (TFF1, TFF2). Results for tetraspanins CD9, CD63, CD81, EV‐specific flotillin, cell markers calnexin and GRP94, as well as culture medium supplement‐derived apolipoprotein A1 (ApoA1) and human serum albumin (HSA). (d) Densitometry of blots shown in (c) normalised to total protein/lane. Significant differences (*p < 0.0332, **p < 0.0021 and ***p < 0.0002) were identified based on two‐tailed t‐test with 95% confidence level. The complete western blot membranes including also control endothelial cells are shown in a separate Western blot supplement

Cryogenic TEM showed characteristic double membrane‐surrounded vesicles (Figure 2a). Super‐resolution microscopy of individual EVs confirmed an immunophenotypically heterogeneous EV preparation with colocalization of tetraspanins CD9, CD63 and CD81. Only 11.7% displayed all three tetraspanins simultaneously and 49.8% were single positive for only one tetraspanin (Figure 2b). Western blot confirmed EV identity regarding minimum information for studies of EVs according to MISEV2018 criteria (Théry et al., 2018). Comparable CD81 and lower CD9 levels were measured in three representative purified EV preparations from therapeutic lots of PLX cells of three individual placentas. Lineage specificity of tetraspanin expression levels was indicated by lower CD81 and higher CD9 levels detected on endothelial cell‐derived control EVs (Figure 2c, Table 1). Loading corresponding protein amounts showed significantly enhanced tetraspanin and flotillin‐1 membrane raft marker signals after sequential TFF. The nonvesicular endoplasmic reticulum lectin, calnexin, and the golgi membrane stacking protein, GRP94, were only found in cell lysates. Apolipoprotein A1 as a marker for plasma‐borne high‐density lipoproteins was enriched and human serum albumin was depleted in TFF2 after enrichment in TFF1 (Figure 2c). Densitometry of EV blots from three independent donors confirmed the results (Figure 2d).

**TABLE 1:** Addressing MISEV 2018 criteria for research with EV material in this study

Nomenclature		
Use of the generic term EV for PLX‐derived EVs		
Collection and storage		
Releasing cell information	cell type and origin	placenta‐expanded stromal cells; placenta samples from three donors
	passaging	< 2
	seeding density	1000 cells/cm^2^
	cell viability	> 95 %
	culture volume	500 ml
	culture vessel	CF4^*^
	oxygen level	5%
Culture conditions	culture medium	α‐MEM, 10% HPL^*^, 5 mM Dipeptiven, 2 U/ml heparin, Pen/Strep
	time of culture	70% confluency / 7 days
	harvesting medium	500 kDA TFF‐filtered culturing medium
	time of cultivation	48 h
	cell count at harvest	40 ‐ 80 × 10^6^/CF4 (8 × 10^4^/ml)
Storage and recovery	conditioned medium	‐80°C, thawing at 37°C
	EV preparations	‐80°C after snap freezing, thawing at 37°C

Isolation	
Differential centrifugation	300 x g for 5 min, SX4750, Allegra X‐15R, Beckman Coulter
	3000 x g for 10 min, SX4750, Allegra X‐15R, Beckman Coulter
Ultrafiltration	TFF^*^ 1600 cm^2^ mPS 300 kDa (500 ml concentrated to 5 ml) (TFF1 EVs)
Purification	1000 ml PBS isovolumetric washing (TFF2 EVs)
Comment	comparison of soluble factors (TFF eluate) with TFF EVs (WB)

Characterization				
		Parameter	Unit	Method
Quantification	Size & concentration	particle number	6 × 10^10^ ‐ 2 × 10^11^ particles/ml	TRPS
		particle size	70 ‐ 420 nm	TRPS
		particle/protein ratio	3.6 ‐ 7.5 × 10^9^ particles/mg	calculated
	Composition	protein content	1 ‐ 38 mg/ml	Bradford / BCA
		lipid content	n.d.	
		RNA content	3 pg/μl	Bioanalyzer RNA 6000 pico
Identity		transmembrane proteins	CD9/63/81, CD44, CD29	WB^*^
		cytosolic recovered in EVs	Flotillin‐1	WB
		protein contaminants	HSA^*^	WB
		lipoprotein contaminants	Apo A1	WB
		secretory pathway	Calnexin, GRP94	WB
		phosphatidylserine	Lactadherin	FL‐triggered FCM^*^ (Cytoflex)
		intact membrane	Calcein staining	FCM^*^ (Cytoflex)
Visualization		high magnification + wide‐field negative contrast TEM^*^		
		high magnification + wide‐field Cryo TEM		
		Super‐resolution microscopy dSTORM (Oxford NanoImager)		

*Abbreviations*: CF4, cell factory four‐layered; HPL, human platelet lysate; TFF, tangential flow filtration; WB, Western blot; HSA, human serum albumin; FL, fluorescence; FCM, flow cytometry; TEM, transmission electron microscope.

### 3.3 Trophic proteins regulating angiogenesis and immunity co‐enriched with PLX‐EVs

Using multiplex bead‐based flow cytometry (Wiklander et al., 2018) for EV surface marker profiling confirmed high expression of tetraspanins CD81/CD63 and reduced CD9 expression. We found medium‐classified fibronectin receptor CD49e/CD29, high/medium extracellular matrix interaction molecules CD44 and NG2, cytokine receptor CD105 (endoglin) and CD142 (coagulation factor III, tissue factor). The majority of haematopoiesis markers were absent on purified PLX‐derived EVs (Figure S3A).

After establishing flow cytometry instrument sensitivity based on silica size marker beads (Figure S3Bi) we defined a gating strategy for nanoparticles sized between ≥ 100 nm up to ≤ 1000 nm. Negative‐control PBS, with and without calcein and lactadherin reagents, showed only minute unspecific reactivity (Figure S3Biii‐iv).

Flow cytometry showed predominantly calcein‐converting cell‐derived EVs (Figure S3Bv). Calcein and lactadherin signal distribution indicated ≥ 96% EVs derived from intact cells (calcein single‐positive) and presumably not apoptotic bodies (lactadherin negative). By back‐gating calcein/lactadherin double‐positive events (Figure S3Bii, PLX EV size gate plot), a minor proportion of apoptotic bodies was apparent in the 500 – 1000 nm size range as described in the literature (Crescitelli et al., 2013) (Figure S3CD).

**FIGURE 3:**
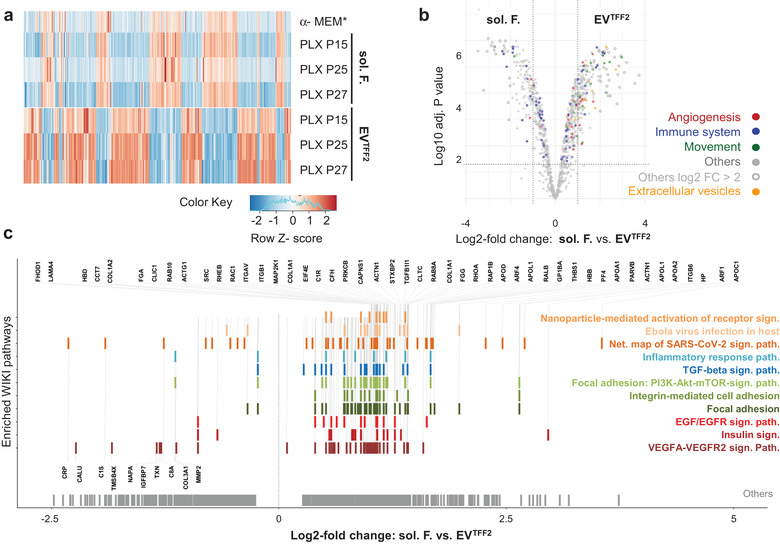
Quantitative proteomics of PLX secretome fractions. (a) Heatmap comparing the proteome of soluble factor factors (sol. F.) and EV^TFF2^ with human platelet lysate‐supplemented defibrinised unconditioned cell culture medium (α‐MEM). (b) Volcano plot comparing protein expression signal significance to levels of enrichment in sol. F. vs. EV^TFF2^. Functional categories according to corresponding GO terms as listed in Table S4 are depicted by color‐coded dots as indicated; additional highly over‐represented proteins marked as open circles (see also Figure S6). (c) Pathway enrichment analysis for significantly differentially detected proteins (*n* = 814, adjusted p < 0.05). Enriched proteins (*n* = 118) and corresponding WIKI pathways highlighted in the same colour. Proteins most significantly (absolute log2‐fold‐change > 1) enriched in soluble factors (bottom left; *n* = 10) and proteins enriched in the EV^TFF2^ fractions (top; *n* = 47) shown. Proteins found in several pathways are shown with one bar with different corresponding colour codes. The remaining differentially detected proteins are highlighted as grey barcode on the bottom (category ‘others’; *n* = 696). Abbreviations: Net., network; path., pathway; sign., signalling

The EV preparation protocol devised in this study allows for high throughput scalable separation of EVs from soluble factors by sequential TFF processing of CM preparations (Figure S5A). Targeted proteomic preanalysis using western blot‐based arrays comparing unconditioned cell culture media vs. CM soluble factor fractions and TFF1 vs. TFF2‐enriched PLX‐EVs, respectively, revealed differences between the four fractions (Figure S5BC). The majority of highly abundant proangiogenic factors was present in both, EV^TFF1^ and EV^TFF2^. We did not find specific factors significantly enriched in purified EV^TFF2^ over EV^TFF1^, using this technology (Figure S5D). We next analysed protein composition of soluble factors and EVs, compared to unconditioned media, to identify proteins uniquely present in different fractions by qualitative label‐free proteomics. Preanalytics confirmed depletion of total protein content in EV TFF fractions by the factor 4.87 – 18.33 accompanied by further 1.34 – 3.30‐fold EV enrichment. A total of 401 ‐ 1168 proteins were detected in different donor‐derived fractions. (Table S3). Only 708 proteins detected in at least two different donor‐derived fractions were selected for further analysis. We identified 258 proteins uniquely present in purified EVs. Of these proteins, 110 were related to either angiogenesis, immune system regulation, cell movement or adhesion. Furthermore, 59 proteins unique to the soluble factors and 18 proteins enriched by TFF were found; 224 proteins were present in all three fractions (Figure S5B). Using differential proteomics with TMT labels, we detected 814 proteins significantly differentially expressed (p < 0.05) with distinct pattern of protein clusters but only minute PLX donor variability (Figure 3a). Complete tandem mass tag proteomics data as uploaded to PRIDE [accession N°.: PXD014572] included 93 significantly enriched immunomodulatory and angiogenic proteins. Proteins significantly over‐represented in EVs after TFF2 were related to immune response modulation, angiogenesis, cell movement and EV marker molecules according to selected corresponding GO terms (Figure 3b; Table S4). Ingenuity pathway analysis revealed several canonical protein signalling pathways differentially over‐represented in EV preparations compared to soluble factor fractions (Figure S6A). Functional and disease categories related to cell angiogenesis, movement and immune response were classified ‘enriched’ in EV proteomes (Figure S6B). An alternative approach searching for proteins corresponding to WIKI pathways related to the main GO categories identified 118 proteins with redundant contribution to vesicle‐related, angiogenic or immune functions. Interestingly, EV^TFF2^ preparations contained 47 proteins significantly over‐represented compared to the EV‐depleted soluble fraction (Figure 3c).

### 3.4 PLX‐EV preparations capture proangiogenic cell‐secreted factors and mediate vascularised skin regeneration in vivo

**FIGURE 4:**
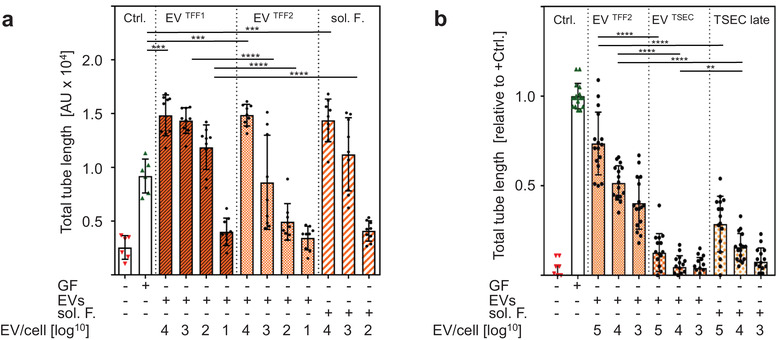
PLX‐EVs capture proangiogenic factors. (a) Angiogenic potential of different PLX secretome fractions as analysed by endothelial network formation in a matrigel assay. Total length of the endothelial networks in the presence of PLX‐EVs at the indicated EV per endothelial cell ratio is shown (e.g., Log10 [3] = 1000:1). Volumes of soluble factors (sol. F.) added to the assay were calculated accordingly corresponding to EV number as described in the methods section. Results pooled from three independent donors (***p < 0.0002, ****p < 0.0001). (b) Separation of EV^TFF2^ from their adjacent proteins by size exclusion chromatography (EV^SEC^) revealed significant loss of proangiogenic function

To validate proteomics, we analysed the different vesicular and vesicle‐depleted PLX secretome fractions for their capacity to stimulate endothelial network formation as a surrogate for angiogenesis. EVs and soluble factors efficiently induced network formation in a dose‐dependent manner. (Figure 4a). Representative images of endothelial network formation are shown per reviewer request (Figure S7). Partial separation of EVs from extravesicular proteins by SEC significantly abrogated proangiogenic function of both EVs and soluble factor fractions, indicating EVs gathering trophic factors (Figure 4b). We next hypothesised that the PLX treatment effects could relate to alleviating therapeutically active proteins in the close vicinity of EVs in a corona‐like fashion.

To question in vivo functionality of the trophic factors in EV^TFF2^ preparations, we took advantage of a historical human cell therapy model for deep skin wound regeneration on immunodeficient mice (Wang et al., 2000). Using this model, we recently demonstrated that platelet‐derived EVs mediate self assembly of human skin and skin organoids (Ebner‐Peking et al., 2021).

**FIGURE 5:**
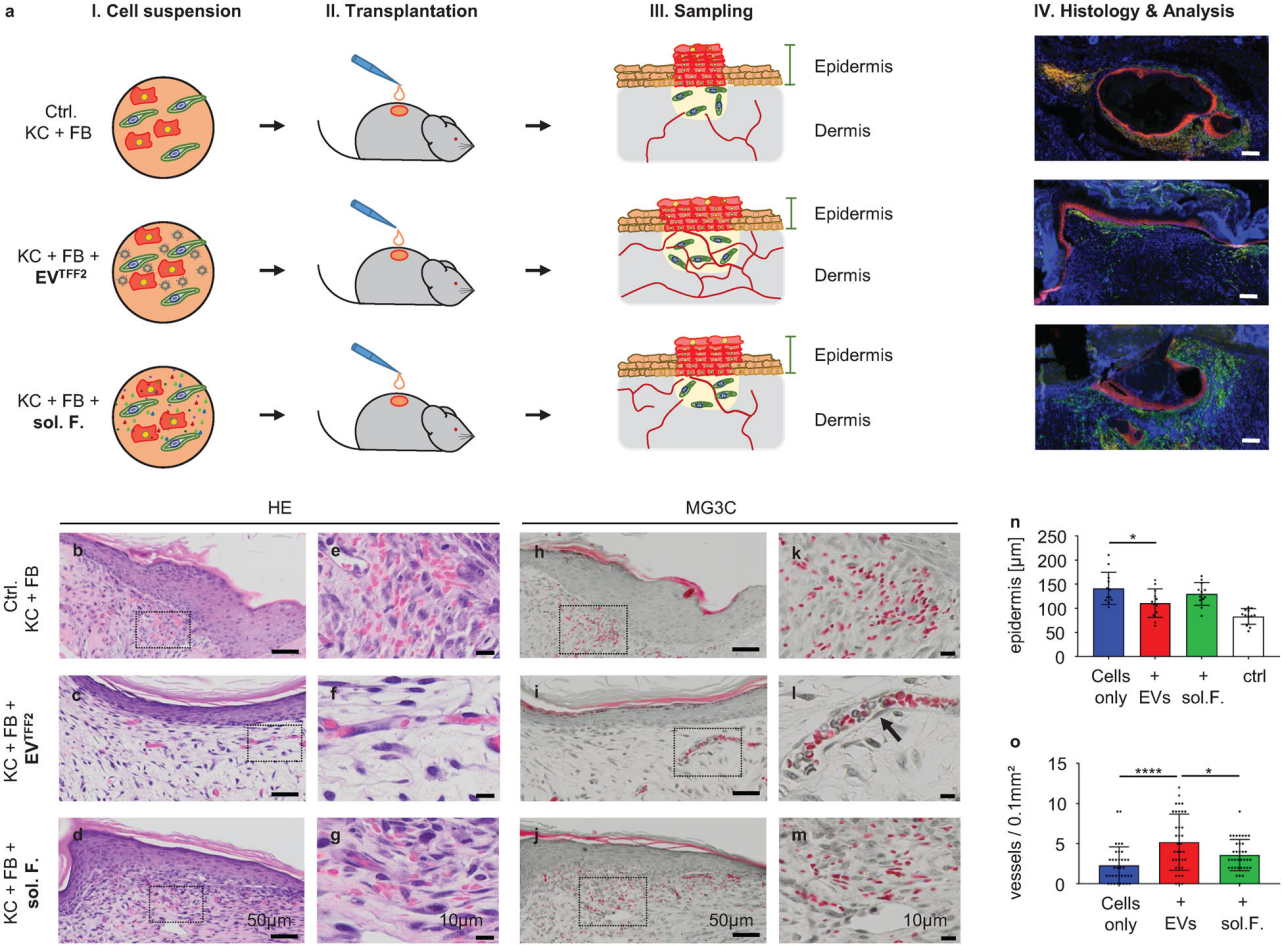
PLX‐EVs promote angiogenesis during skin regeneration in vivo. (a) Graphic illustration of the skin cell transplantation strategy testing EV vs. soluble factor (sol. F.) function during in vivo full thickness skin wound regeneration (Ebner‐Peking et al., 2021). Keratinocyte + fibroblast (KC + FB) cell suspension grafts, transplanted in the absence or presence of either TFF2‐isolated PLX‐EVs (+EV^TFF2^) or sol. F. as described in the methods section (step I + II). Human skin biopsy sampling (III) and representative immunohistochemistry (IV; antihuman CD44, red; antihuman vimentin, green). (b‐m) Histology of day 14 post grafting. Hematoxylin and eosin (HE) and Masson Goldner trichrome (MG3C) staining confirmed the layered cell organization into epidermis and dermis. Dermal analysis showed vessel enrichment predominantly when supporting the cell grafts with EV^TFF2^. Serum only and sol. F.‐driven cell transplants revealed limited murine vessel sprouting and tissue haemorrhage. Vessels were stabilised by pericytes (arrow in L). Dotted boxes in (b‐d; h‐j) indicate magnified areas in (e‐g; k‐m). (n) Quantification showing significantly increased epidermal thickness (Cox & Mann, 2008) in cell grafts in the absence of EVs^TFF2^ in transplants of KC+FB ‘cells only’ or KC+FB transplanted in the presence of sol.F:, compared to control human skin (ctrl). (o) Vessel density in grafted dermis. Mean ± SD results (n, o). One Way‐ANOVA, multiple comparison of four biological and three technical replicates (*p < 0.05, ****p < 0.0001)

Transplantation of human keratinocytes plus fibroblasts (Ebner‐Peking et al., 2021) in the presence of TFF2‐enriched PLX‐EVs resulted in rapid human skin regeneration with pronounced early vascularization after 2 weeks already. EV‐deprived PLX‐derived soluble factors supported skin regeneration comparable to control transplants. Remarkably, only transplants in the presence of EVs showed patent vessel ingrowth whereas transplants without EVs showed significantly less sprouting murine vessels and pronounced red cell extravasation (Figure 5).

Engrafted human skin showed appropriate stratification and enhanced engraftment for the EV‐treated animals compared to controls (Figure S8). We observed more patent epidermal and dermal organization (Figure 5a‐iv) in addition to a less hypertrophic epidermis (Figure 5n) and higher in‐sprouting murine vessel density (Figure 5o). Human keratinocyte plus fibroblast transplants in the absence of EVs (‘cells only’ or cells + soluble factors without EVs) showed submerged human skin structures due to murine skin contraction (Figure 5a‐iv). We speculated that extended stability and/or tissue protease resistance of proangiogenic factors in the vicinity to EVs could mediate such differences. To address this issue, we subjected EV^TFF2^ and EV‐depleted soluble factors to protease treatment before measuring trophic protein persistence by antibody‐based array profiling. EVs protected some but not all secreted factors including matrix metalloproteinase‐8 (MMP‐8), angiogenin, serpins, coagulation factors, angiopoietin and vascular endothelial cell growth factor (VEGF) from degradation. However, functional testing in an angiogenesis assay showed significantly reduced vascular network formation after protease treatment of both EVs as well as soluble factors (Figure S9).

### 3.5 PLX‐EV preparations modulate T cell proliferation and target immune cells

**FIGURE 6:**
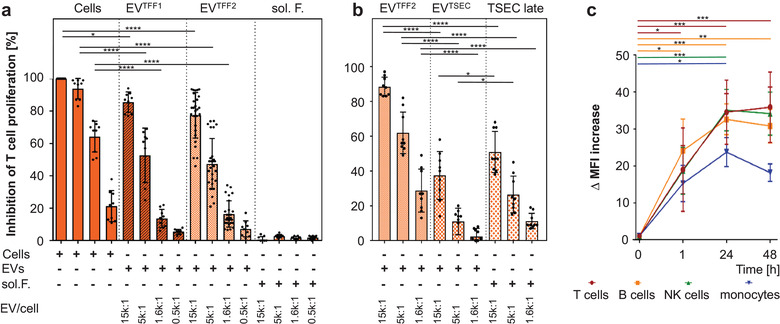
Immunomodulation and cell signalling by EVs: (a) Inhibition of phytohemagglutinin (PHA)‐induced T cell proliferation by PLX stromal cells, EV^TFF1^ and EV^TFF2^ as compared to soluble factors (sol. F.) added as indicated in limited dilution at defined ratio to mononuclear leukocytes. The percentage of inhibition was calculated relative to the maximum proliferation induced by PHA. Pooled results of three independent donors measured at day four. The first four bars show inhibition of T cell proliferation in a dose‐response to decreasing number of PLX:T cells of 1:1, 1:3, 1:9, 1:27, from left to right. (b). Assay format as used in (a) but testing the inhibition of T cell proliferation by size‐exclusion‐purified EV^TSEC^ and their former corona separated by size‐exclusion chromatography, compared to parental EV^TFF2^. (c) Peripheral blood mononuclear cells were incubated with bodipy‐labelled EV^TFF2^ at predetermined ratio of 1:5000 for one, 24 and 48 h as indicated. The bodipy signal was located to CD3^+^ T cells, CD19^+^ b cells, CD56^+^ NK cells and CD14^+^ monocytes by polychromatic flow cytometry. Pooled results from three independent donors performed in triplicate were analysed (in a‐c). Statistical analysis was done in Graph‐Pad Prizm 7.03 using one way ANOVA analysis with Sidak correction for multiple samples (** p < 0.002; *** p < 0.0002; **** p < 0.0001)

To study immunological aspects, we tested the potential of different PLX secretome fractions, compared to parental PLX cells, to inhibit mitogen‐driven T cell proliferation. PLX cells and their EVs significantly inhibited T cell proliferation in a dose‐dependent manner with PLX cells reaching almost 100% inhibition at a 1:1 ratio. PLX‐derived soluble factors did not inhibit T cell proliferation in the absence of EVs (Figure 6a). Interestingly, further separating EV^TFF2^ by SEC from their adjacent protein fraction divided the immunomodulatory capacity into EV^TSEC^ and their proteins, both inhibiting T cell proliferation significantly less than EV^TFF2^ (Figure 6b). At a mechanistic level, time‐dependent significant uptake of fluorescently labelled EV^TFF2^ was observed in four major immune cell types by multicolour flow cytometry (Figure 6c).

Confocal microscopy of sort‐purified cells confirmed cytoplasmatic localization of EV^TFF2^ after 24 h (Figure S10A). Sort‐purified T cells and monocytes were selected to determine intracellular signalling of EVs in target cells. A differential signalling signature was observed with over‐representation of phosphorylated cyclin‐dependent kinase inhibitor p27 and glycogen synthase kinase‐3, regulating cell cycle and signalling, respectively (Figure S10B).

### 3.6 Evidence for the EV corona

**FIGURE 7:**
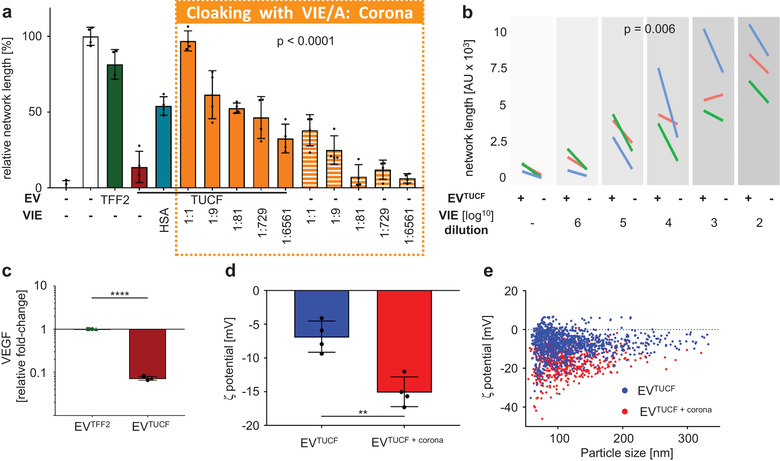
EV protein corona analysis: (a) Endothelial cell network formation comparing EVs^TFF2^ with ultracentrifuged, that is, corona‐depleted EVs (EV^TUCF^) and free growth factors and growth factor corona‐cloaked EVs. Representative experiment, quadruplicates. (b) Dose dependent endothelial cell network formation in the presence (+) or absence (‐) of EV^TUCF^ in an 8% human albumin solution containing VEGF, IGF and EGF (VIE/A) (*n* = 3; individual additional experiments colour coded in red, green, blue, and covering a broader dose range than in (a)). Statistics were calculated using a linear mixed model with treatment as fixed effect and dilution as random effect (a, b). (c) Fold‐change of VEGF concentration comparing EV^TFF2^ to EV^TUCF^ (mean ± SD, *n* = 3, T‐test, p < 0.05). (d) Zeta potential of EV^TUCF^ in the absence or presence of a re‐established protein corona (+ corona) measured in quadruplicates by TRPS. Mode of distribution was used for statistical analysis (T test; **p < 0.01). (e) Zeta potential distribution vs. individual EV size (pooled dataset; *n* = 4)

To address the question whether a protein corona determines EV function, as observed previously for various synthetic and inorganic nanocarriers, we compared EV^TFF2^ (i.e., corona‐bearing) versus ultracentrifugation‐purified EVs (i.e., corona‐depleted) for their proangiogenic potential. Ultracentrifugation resulted in significant loss of EV‐mediated vascular network formation. We selected VEGF, IGF and EGF dissolved in albumin solution (VIE/A) for re‐establishing an artificial corona around ultracentrifugation‐purified EVs. The VIE/A‐cloaked EVs significantly re‐established their proangiogenic potential in a corona protein‐dose‐dependent manner. These corona‐bearing EVs were significantly more efficient than respective doses of VIE/A in the absence of EVs over a large log‐range of concentrations. Adding human serum albumin to EVs without VIE led to minor but significant improvement of EV function (Figure 7ab). Significant 10‐fold relative depletion of VEGF, as reference angiogenesis factor, was confirmed by enzyme‐linked immunoassay after ultracentrifugation of EV^TFF2^ (Figure 7c).

**FIGURE 8:**
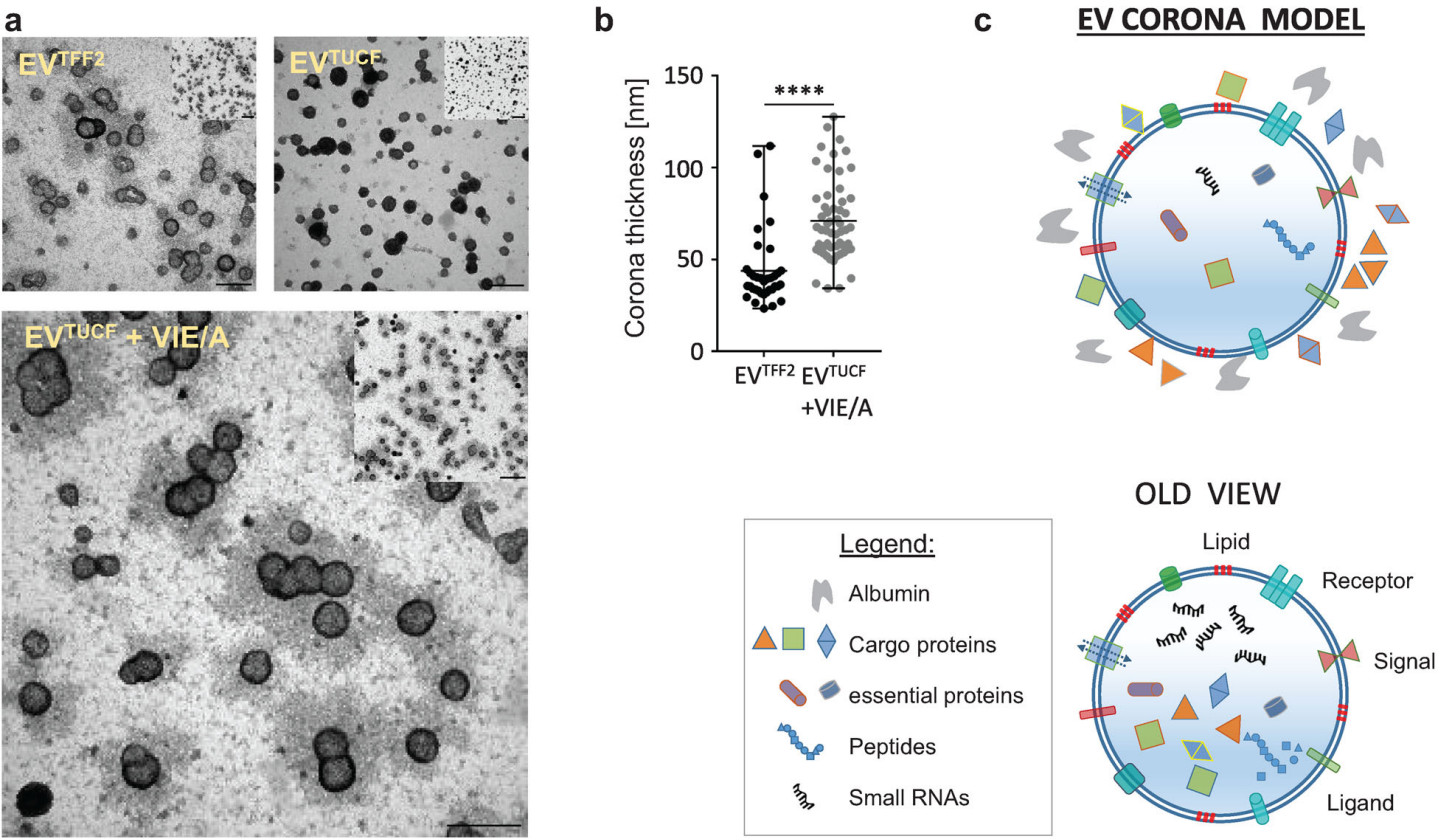
EV protein corona location: (a) Negative contrast TEM images of PLX EVs purified with tangential flow filtration (TFF2), depleted protein corona after TFF followed by ultracentrifugation (TUCF) and with protein corona re‐established by defined factors VEGF+ IGF+ EGF in albumin solution (VIE/A). Scale bar 250 nm, small insert showing overview image with scale bar 500 nm. (b) Corona thickness based on negative contrast halo measurements as shown in detail in Figure S12. We analyzed *n* = 36 EV^TFF2^ and *n* = 56 EV^TUCF^+VIE/A; Mann–Whitney unpaired sample test, ****p > 0.0001. (c) New EV corona model and current/old view on EV structure (modified from (Kalluri & Lebleu, 2020) and www.exosome‐rna.com/evpedia)

Polystyrene nanoparticle surface properties are known to determine corona properties (Lundqvist et al., 2008). We therefore decided, in an analogy between nanoparticles (Figure S11) and EVs, to measure zeta‐potentials of EV preparations in the hypothesised absence or presence of a corona (Figure 7d and e). To visualise the postulated protein corona around PLX‐EVs we took advantage of conventional negative‐contrast TEM. EVs^TFF2^ showed a halo around vesicles, suggesting a corona, that was absent after ultracentrifugation. The halo appeared again after cloaking ultracentrifugation‐purified EVs with VIE/A indicating corona formation (Figure 8a). These pictures were highly reminiscent of previous results showing the prototypic polystyrene nanocarrier corona (Kokkinopoulou et al., 2017). Visualization of the nanoparticle corona was done previously by negative‐contrast TEM showing between 15 nm hard corona to > 50 nm soft corona diameters depending on the ultracentrifugation washing procedure (Kokkinopoulou et al., 2017). Based on these results we suggest a new model that considers the nanoparticle corona on cell‐derived EVs (Figure 8b).

## 4 DISCUSSION

This study was initiated by the hypothesis that EVs contribute to the mode of action of PLX stromal cell therapy aiming to cure peripheral artery disease, with a certain focus on angiogenesis and immunomodulation being the proposed operative mechanisms. Our major unexpected finding was, by thoughtful observation, that PLX‐EVs bear a functional corona. In the course of the study, we observed that further purification of functional TFF‐EVs by both, SEC and UCF, affected EV functions. Our strategy to confirm the provocative hypothesis that this relates to EV corona removal during the purification process is discussed in detail below. We could reconstitute the EV function by cloaking nonfunctional EV^TUCF^ with an artificial corona comprising three bioactive proteins in albumin (VIE/A) and visualise this process by negative contrast TEM, indicating corona removal and reformation. As a proof of concept, we visualised the corona formation by super‐resolution microscopy using fluorescent albumin and fluorescent VEGF. During the review process of this study, we also demonstrated in a separate publication (Gomes et al., 2022) that loss of function of EVs after SEC can be fully reverted by adding the SEC late fraction. Corona formation was indicated by means of fluorescent albumin as a model protein (Gomes et al., 2022).

The canonical view on cell‐derived EVs displays a cargo‐carrying core enveloped by a receptor‐bearing double membrane and a rich internal cargo (Figure 8c). This basic structure was sufficient to explain many EV functions during cell‐to‐cell communication and built the basis for designing specialised EV products as next‐generation drug delivery carriers (Herrmann et al., 2021). During the review process of this study, the Buzas group demonstrated for the first time that a protein corona can be formed on the surface of EVs in blood plasma (Tóth et al., 2021). Our observation that cell‐derived EVs can acquire a functional corona even in advance of plasma conversation, together with our demonstration a proangiogenic functional corona can be created and visualised by cloaking ultracentrifugation‐purified EVs with VIE/A, adds another level of complexity to EV biology. The landmark study by Tóth et al. (2021) and our study highlights that biological cell‐derived EVs can be subject to corona formation, a phenomenon previously thought to be restricted to synthetic nanoparticles. The precise molecular mechanisms leading to corona formation and release of the functional EV corona components at the target cells need to be disclosed. We can just speculate that corona‐bearing EVs can also form a nanoscale synapse, as proposed for other nanostructures (Dawson & Yan, 2021), when executing their function.

In this study we started by characterizing PLX‐EVs based on MISEV2018 guidelines (Théry et al., 2018) and found a heterogenous EV population regarding tetraspanin distribution. Taking advantage of the straightforward separation of soluble factors from EVs out of conditioned media by stepwise TFF (Figure S2), we were able to perform in‐depth proteomic analysis. We demonstrate that proteomes in soluble factor as compared to EV fractions differ significantly, compatible with an enrichment of certain proteins in close vicinity to EVs. Despite these differences, both, TFF‐purified PLX‐EV fractions as well as the soluble factors, induced vascular network formation in a dose‐dependent manner in vitro. This could be explained by the fact that several proteins involved in vascular remodelling, for example, serpins, platelet factor‐4 (PF‐4), thrombospondin‐1 (TSP‐1) and angiogenin, were present in the soluble factor fraction as well as in EV preparations. Provided that a comparable ‘soluble factors plus EV’ secretome pattern also occurs in vivo, our results would indicate that PLX stromal cell‐derived EVs may be considered to contribute to the therapeutic effect. In our humanised mouse model, TFF‐EVs were superior to PLX‐derived soluble factors in mediating angiogenesis and wound healing. We may speculate that EVs can act over longer periods of time beyond rejection of the allogenic cell therapy and degradation of secreted factors in patients. A precise assignment of the different aspects of vascular and tissue remodelling as well as wound healing induced by PLX cells or their soluble and vesicular secretome fractions requires additional research that is currently underway.

So far, the therapeutically active cell secretome was considered to comprise trophic secreted factors in addition to EVs (Witwer et al., 2019). In an attempt to further purify the PLX‐EVs we observed a loss of proangiogenic EV function that could not be explained by a shift of angiogenic activity into the protein‐rich late EV fractions, termed soluble factors. EV corona formation offers one attractive way for explaining this observation.

We identified immunity‐related proteins, in addition to angiogenesis factors, as being over‐represented particularly in purified PLX‐EVs. PLX, like other stromal cells, have immunomodulatory capacity (Winkler et al., 2018). In contrast to angiogenesis, dose‐dependent inhibition of T cell proliferation was observed in our study only with PLX cells and their corona‐bearing TFF‐EVs but not by TFF‐separated soluble factors. Understanding the relative contribution of cells vs. soluble factors or EVs to therapeutic efficacy will be a major challenge on the way toward rational design of efficient cell‐derived and EV‐based therapies. Our observation that SEC‐based purification reduced the immunomodulatory capacity of EVs could be interpreted as additional evidence for EV corona removal by SEC, because the SEC late fractions, presumably containing immunomodulatory corona proteins, showed significant immunomodulatory activity that was not observed with TFF‐derived soluble factors. Additional experiments are required to test this hypothesis.

In technical disciplines nanoparticles are traditionally ultracentrifuged for at least 1 h to remove their corona (Kokkinopoulou et al., 2017). Procedural parameters, ion composition and concentration as well as pH can impact corona composition (Ilett et al., 2020). In the biomedical EV field, the established view on UCF and SEC considers both techniques to ‘just’ separate trophic soluble factors from EVs but did not consider the existence of an EV corona (Monguió‐Tortajada et al., 2019). Interestingly, Tóth et al. (2021) also showed that the EVs purified in their study by SEC lost approximately 75% of proteins compared to differentially centrifuged EVs tested by nonquantitative proteomics in advance of SEC. Based on our results we speculate that this could also indicate corona removal by SEC. We are aware that these observations need to be challenged by other research groups to fully understand the mechanistic details of how isolation procedures interfere with EV function.

This study has certain limitations. The precise position of EV‐associated proteins and their binding kinetics still need to be determined. During the review process, we performed preliminary experiments addressing some of these points. Using fluorescent albumin as a model protein we found accumulation of fluorescence in a corona‐like manner in close vicinity to the surface of tetraspanin colabelled EVs within 1 h. In a separate set of experiments, we fluorescently labelled VEGF and observed corona‐like location, although to a lesser extent than found for albumin. The labelling process did not change EV counts nor did it show evidence for aggregate formation (Figure S13). The kinetics were highly reminiscent of those described for corona formation on synthetic nanoparticles (Feiner‐Gracia et al., 2017). Questions regarding soft vs. hard corona composition and function also need to be addressed for EVs. The fact that we did not observe fibrin aggregates as shown by Tóth et al. (2021) during corona formation in fibrinogen‐containing plasma can be considered to be due to the lack of fibrinogen in our protein‐rich but fibrin‐depleted media. Additional studies are clearly required to better understand the biology and function of the EV corona. Tóth et al. (2021) demonstrated the impact of the EV corona on the cytokine secretion profile of monocyte‐derived EVs. We found angiogenic activity in addition to immunomodulatory function. Additional preliminary experiments revealed that blocking VEGF function on EVs only partly reduced their angiogenic potential. We also found that a reduced corona comprising single growth factors was less efficient than VIE/A (Figure S14). These experiments build the basis for further mechanistic studies. Questions regarding corona stability, protein turnover and particularly regarding the fate of a preformed corona during plasma conversation need to be addressed. In addition, the impact of different well‐established isolation procedures like SEC and UCF needs to be revised. The better understanding of EV corona functions will hopefully contribute to the development of better diagnostic and therapeutic strategies.

## CONFLICT OF INTEREST

The authors have declared no conflict of interest.

## Supporting information

Supporting Information
