# Supplementary material for: A functional corona around extracellular vesicles enhances angiogenesis, skin regeneration and immunomodulation
Source: J Extracell Vesicles. 2022 Apr 9;11(4):e12207. doi: 10.1002/jev2.12207 (PMC8994701; doi:10.1002/jev2.12207)
Supplement: Supplementary file 1 — Supporting Information [file JEV2-11-e12207-s001.pdf]

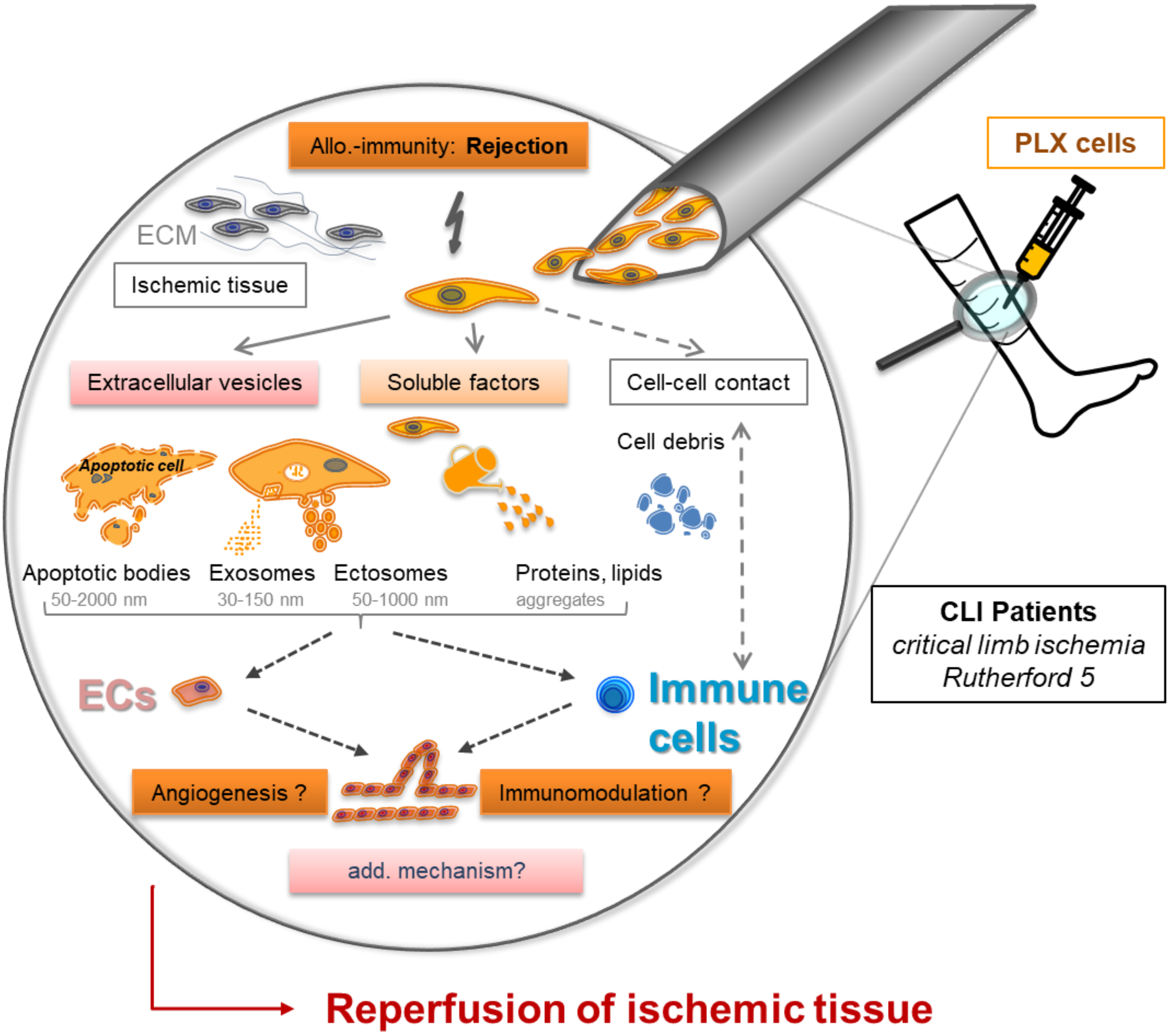

Figure S1. Hypothetic mode of action of allogeneic PLX stromal cells. Allogeneic placental-expanded (PLX) cells are considered to be rejected by the host immune system after local injection. The mode of action may include a temporary direct (cell-cell contact) or indirect stimulation of endogenous (endothelial or perivascular or interstitial) progenitor cells as well as immune response modulation by various types of secreted factors eventually resulting in reperfusion of the ischemic tissue and ideally wound healing. In this study, we focused on extracellular vesicles (EVs) and their protein cargo, compared to secreted soluble factors as potential mediators of PLX's biologic activity.

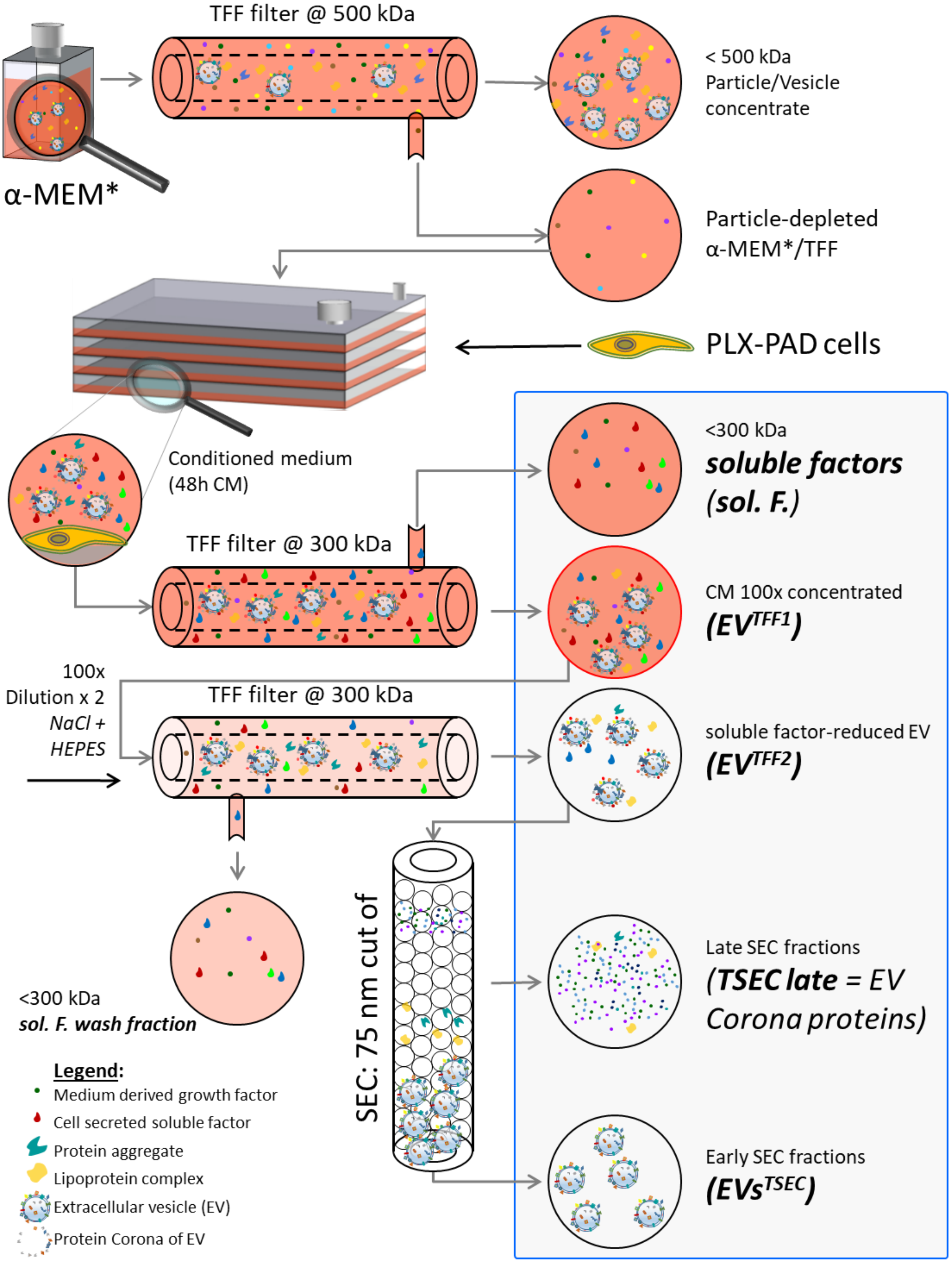

Figure S2: Schematic workflow of large-scale EV production from PLX cells. Cell culture medium α-MEM was supplemented with 10% pooled human platelet lysate (HPL) and fibrinogen-depleted (α-MEM*) before filtration through a 500 kDa cut-off membrane. 70% confluent PLX cells were cultured in particle-depleted α-MEM*/TFF for one to three 48-hour periods to obtain conditioned medium (CM). PLX-EVs were enriched 100-fold by TFF concentration (TFF1s) and separated from soluble factors (sol. F.). To remove remaining soluble factors/proteins, this crude concentrate was washed with twice the initial start volume to yield protein-TFF2 EVs separated from remote soluble factors. Size exclusion chromatography (SEC) was used to deplete EV-co-enriched soft corona proteins for selected experiments.

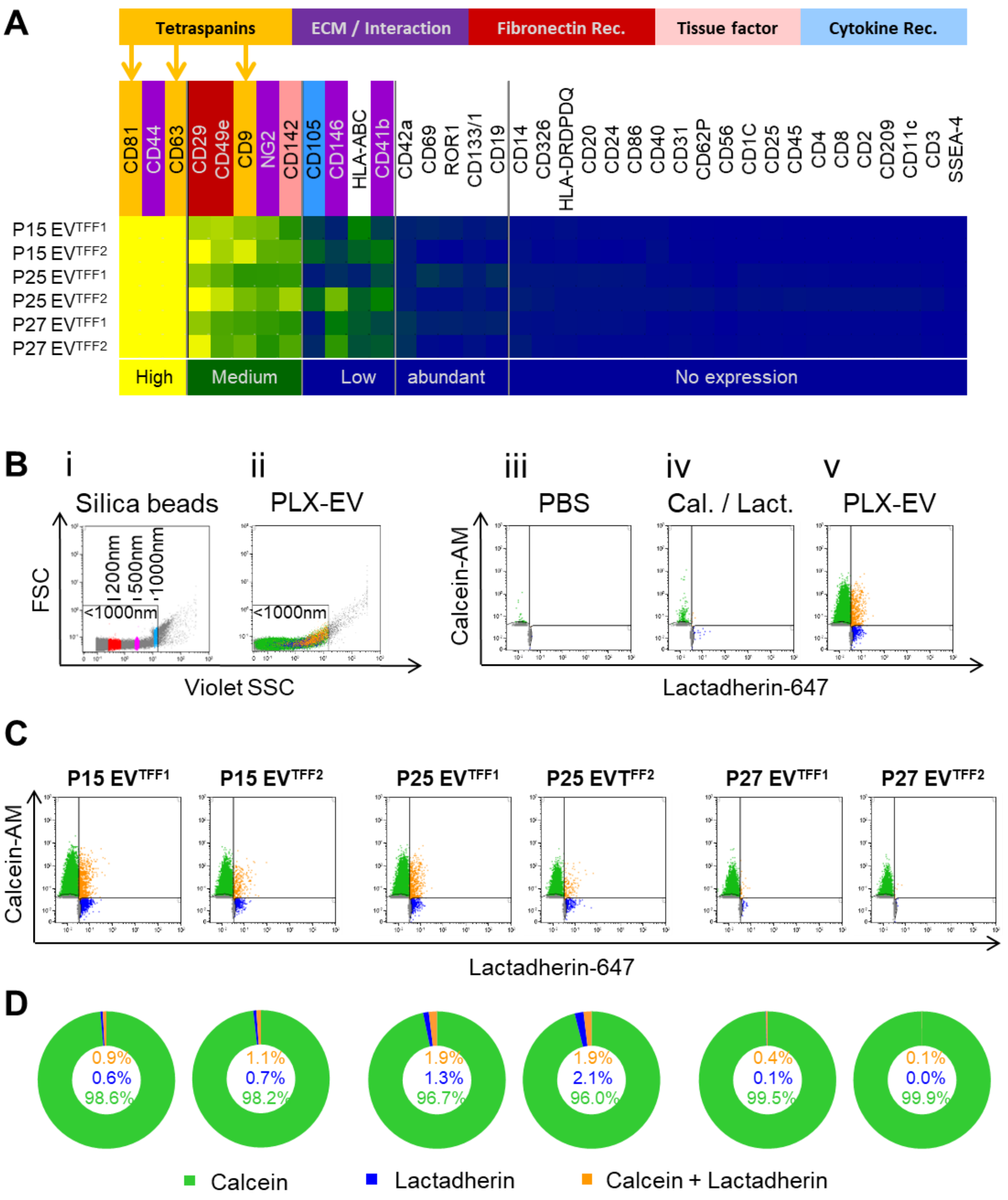

Figure S3: Antibody-based EV marker profiling indicated distinct address code and low abundance of apoptotic bodies. (**A**) Heat map showing mean surface marker expression of EV^{TFF1} vs. EV^{TFF2} from three individual donors (P15, P25, P27) as determined by bead-based multiplex flow cytometry (MACSPlex). (**B, i**) Fluorescent silica beads (Kisker) were used to adjust small particle resolution in flow cytometry and to set a size gate < 1,000 nm for EV detection. (**B, ii**) Dot plot showing size distribution of a representative PLX-EV sample. (**B, iii**) Dot plots showing fluorescence background of unstained PBS control, (**B, iv**) calcein-AM and lactadherin-Alexa Fluor 647 stained PBS control without EVs added (Cal. / Lact.) and (**B, v**) representative double-stained PLX-EV plot showing the distribution of calcein$^+$ and lactadherin$^+$ events using a dual fluorescence trigger. (**C**) Determination of calcein$^+$ EVs and lactadherin$^+$ presumably apoptotic bodies in EV^{TFF1} vs. EV^{TFF2} preparations. Dual fluorescence triggering-derived dot plots based on the gating strategy shown in (B) and color code as in (D) from three independent donor pairs of EV^{TFF1} vs. EV^{TFF2} preparations from short-term PLX cultures of placenta lots P15, P25 and P27, respectively. (**D**) Pie charts depicting marker distribution of the positive events. Negative events were excluded from analysis because current technology does not permit precise discrimination between electronic noise and non-fluorescent unstained EVs or other undetermined non-particulate non-fluorescent signals notably in the size range of 100 nm and below[49].

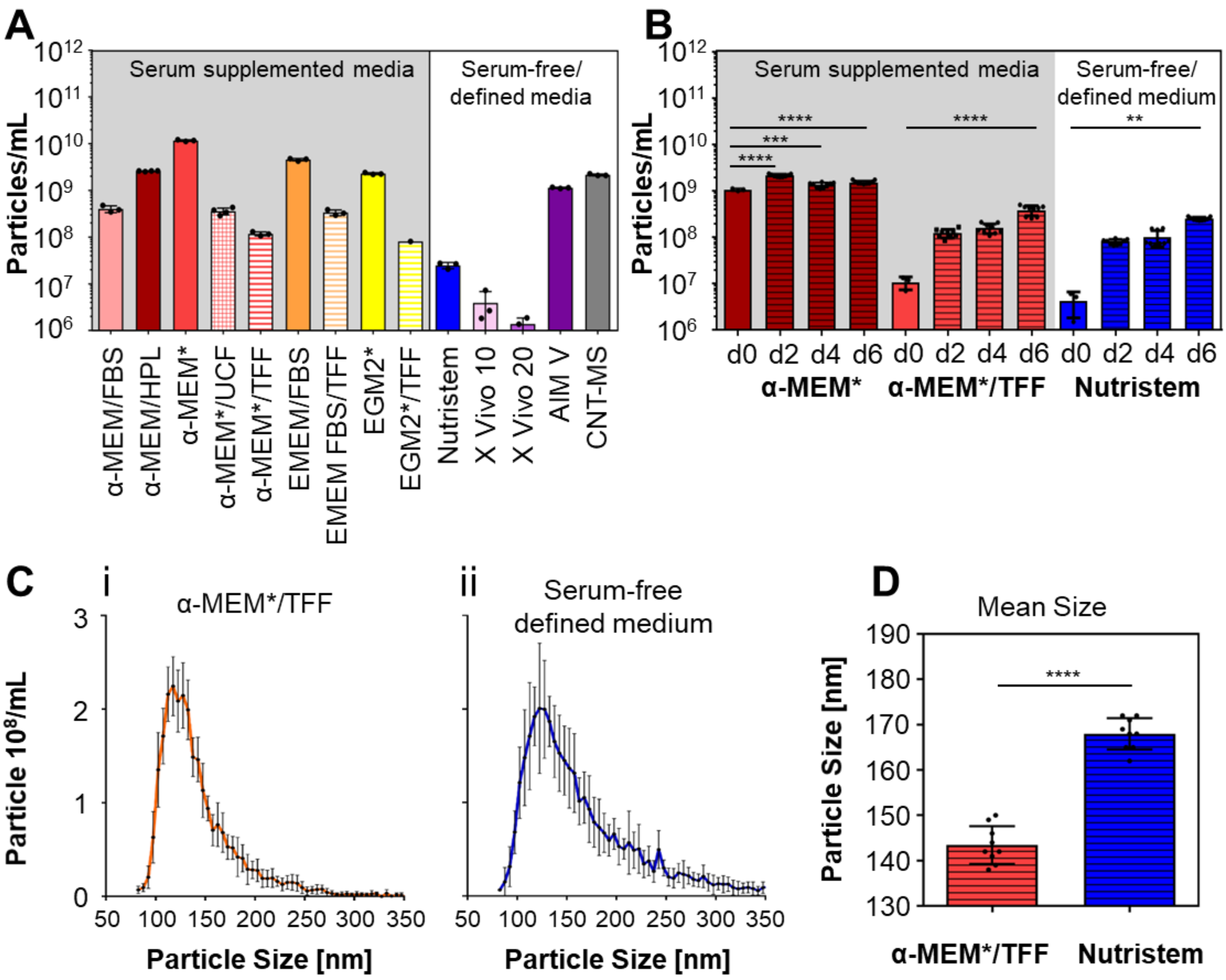

Figure S4: Physically defined media are required for cell-derived EV characterization. (**A**) Particle content of selected cell culture media with (left) or without serum (right) was measured by tunable resistive pulse sensing (TRPS). Media details are given in the methods section. (**B**) Comparison of the particle count in fresh and conditioned media after six-day placental stromal cell culture (PLX+). Particle concentrations were measured from three independent donors in four different media. (**C**) Time course of EV production in α-MEM*/TFF (*see* ***Fig.2***) was compared to serum free defined medium. TRPS analysis was performed for three independent donors in triplicates over six days. (**D**) TRPS measurement of PLX stromal cell-secreted EVs showed (**iii**) significantly different particle size distribution in (**ii**) defined serum-free vs. (**i**) platelet lysate-supplemented media (TFF pre-depleted). Triplicate measurements from one representative donor are shown (i, ii). Data from three donors were analyzed in triplicate (iii).

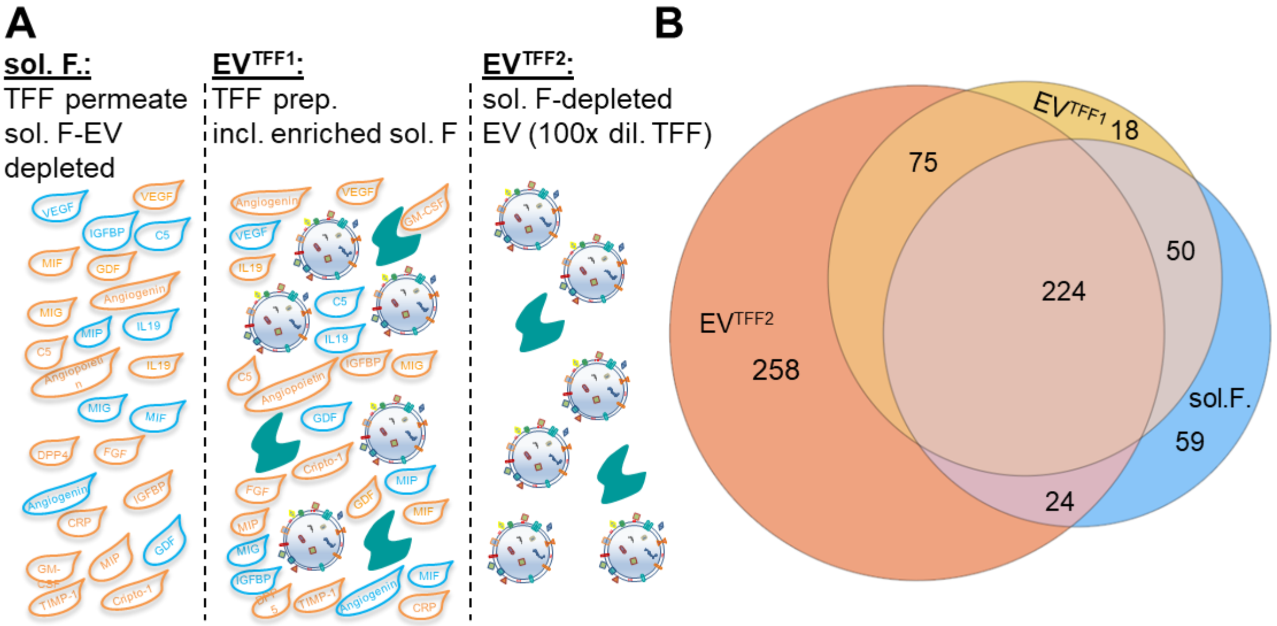

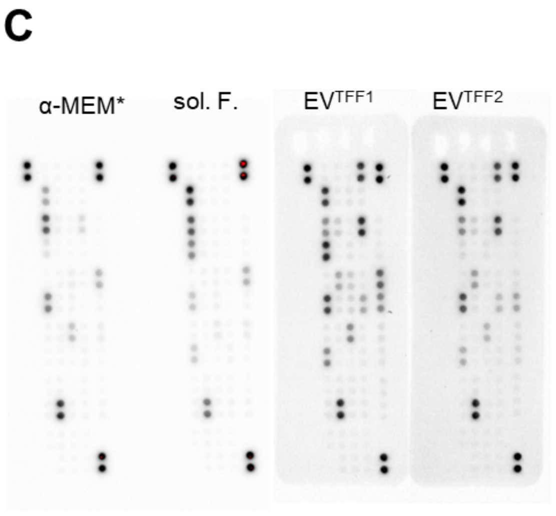

D

Proangiogenic factors	α-MEM*	sol. F.	EV^{TFF1}	EV^{TFF2}
Serpin E1 PAI-1	0,230	0,675	0,730	0,859
Platelet Factor 4 (PF4) CXCL4	0,563	0,355	0,757	0,787
Thrombospondin-1 /TSP-1	0,358	0,139	0,669	0,599
Serpin F1 PEDF	0,472	0,486	0,355	0,358
Coagulation Factor III (TF) (CD142)	0,001	0,001	0,699	0,613
Pentraxin 3 (PTX3) TSG-14	0,001	0,005	0,225	0,261
Angiopoietin-1	0,013	0,001	0,363	0,244
Endoglin (CD105)	-0,001	0,000	0,243	0,181
uPA	0,001	0,122	0,397	0,225
DPP IV/CD26	0,079	0,002	0,798	0,652
IGFBP-3	0,134	0,020	0,367	0,160
TIMP-1	0,037	0,353	0,854	0,011
Angiogenin	0,203	0,204	0,562	0,036
IGFBP-1	0,005	0,007	0,090	0,006
IGFBP-2	0,003	0,005	0,070	0,003
MMP-9	0,003	0,006	0,050	0,016
HGF	0,001	0,002	0,026	0,006
Serpin B5 Maspin	0,008	0,011	0,002	0,025
Angiopoietin-2	0,033	0,045	0,011	0,041

Figure S5: Proteomic composition of PLX secretome fractions. (**A**) Illustration of different fractions separated by TFF. A graphic symbol legend is shown in Figure 1. (**B**) Venn diagram showing the overlap and differences in proteins identified by label-free proteome analysis in the different secretome fractions (soluble factors, sol. F.; TFF1; purified EV, pur. EV) from three donors in two independent experiments. (**C**) Antibody array-based analysis of proangiogenic factors in the different secretome fractions (derived from one representative donor). (**D**) Heat map representation of corresponding quantitative analysis of PLX secretome fractions as indicated. Numbers represent relative luminescence units.

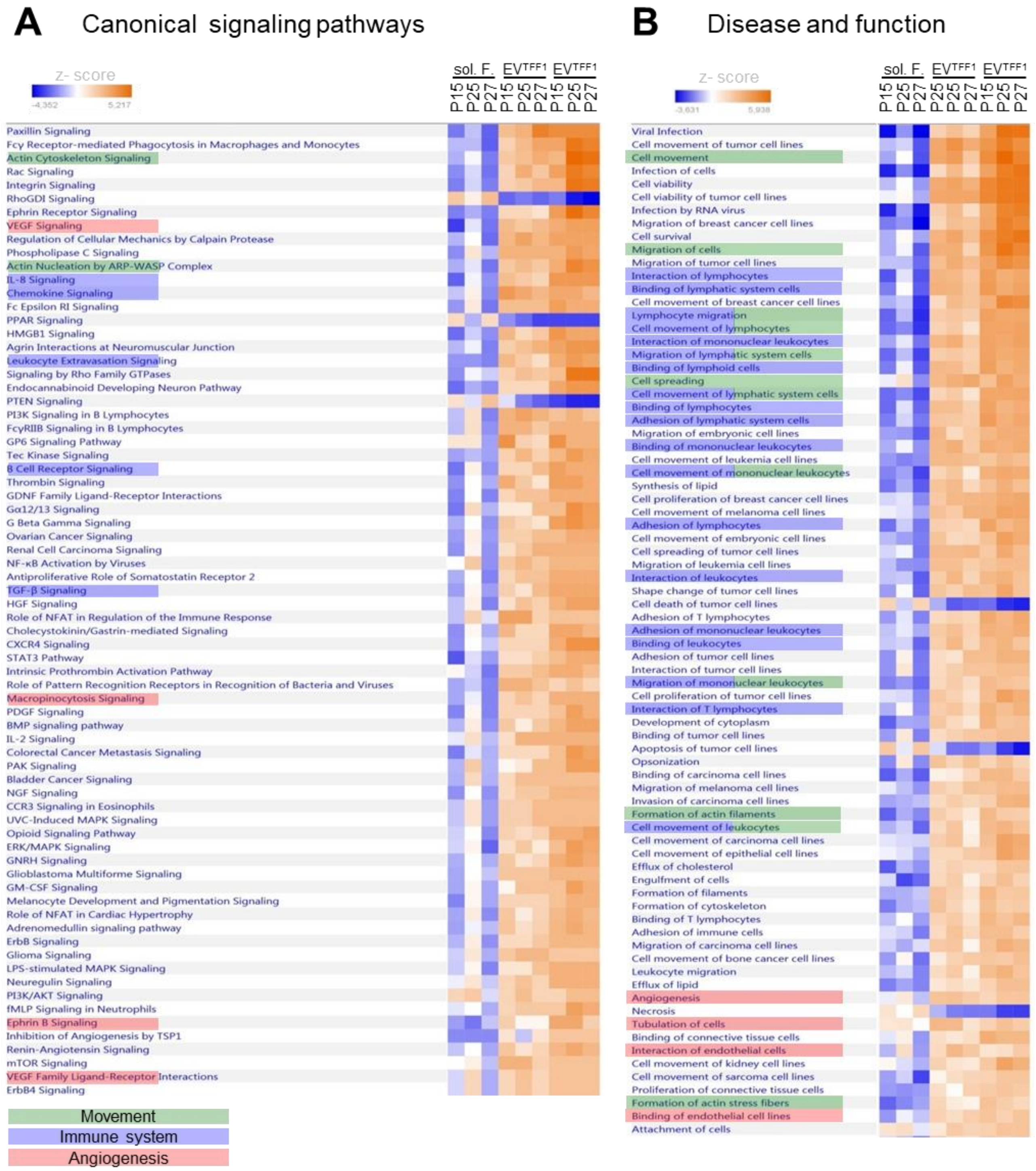

Figure S6: Ingenuity pathway (IPA) enrichment analysis of quantitative proteomics of the PLX secretome fractions. (**A**) Canonical signaling pathways differentially abundant in EVs versus soluble factors (sol.F.) are shown in a heatmap for three individual donors (P15, P25, P27) analyzed. Pathways related to movement, the immune system and angiogenesis are colored green, blue and red, respectively, as indicated**.** (**B**) Disease and function categories from the ingenuity database with the greatest differences in corresponding protein abundances between EVs and soluble factor fractions are shown in a heatmap using the same color code for highlighting pathways.

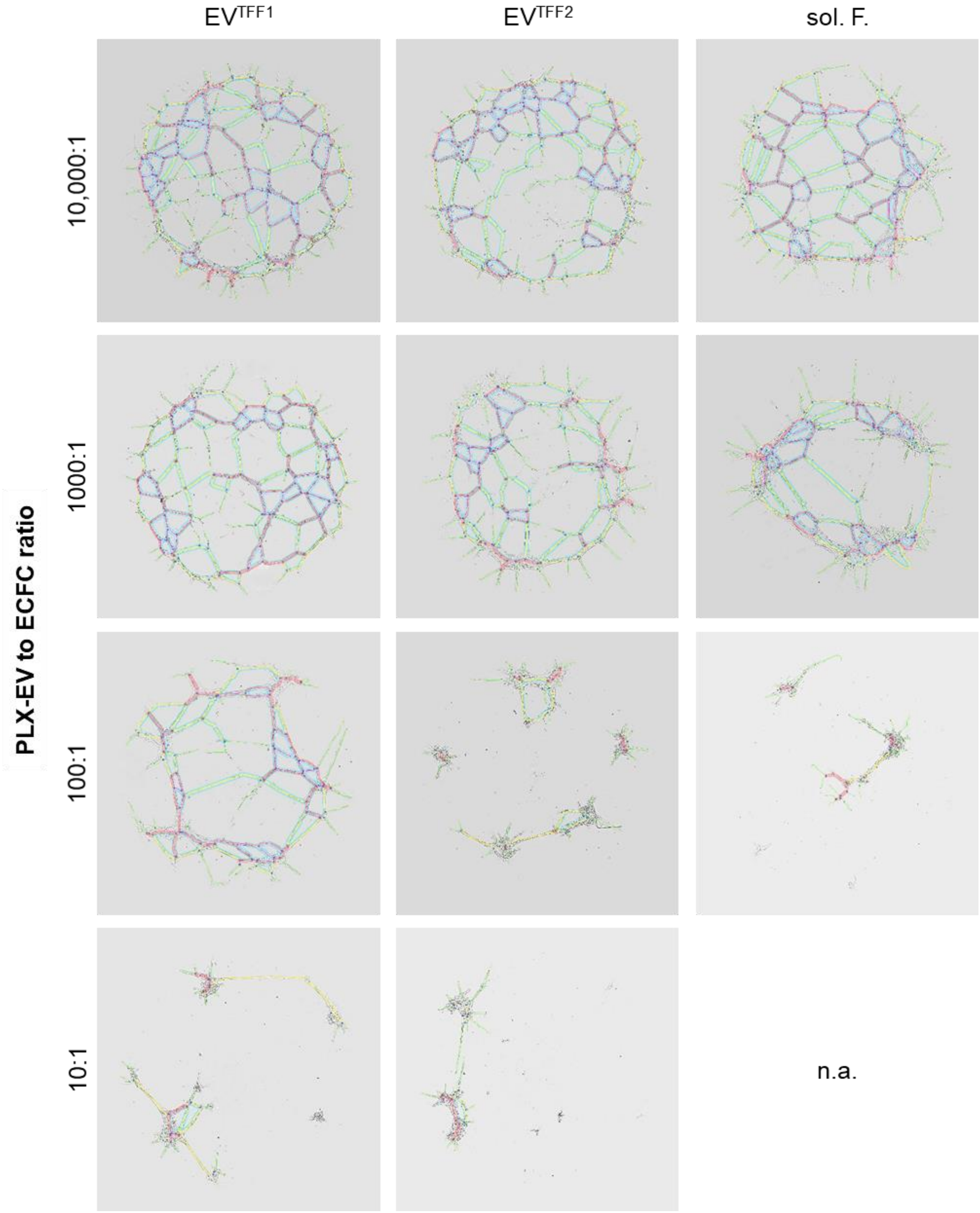

Figure S7: Endothelial network formations. Representative examples of endothelial cell networks (shown in figure 4) formed after treatment with different PLX secretome fractions. Phase contrast images are overlaid with the networks recognized by imageJ angioanalyzer plugin shown with its color code for master segments, segments and branches. Not analyzed, n.a., for technical reasons.

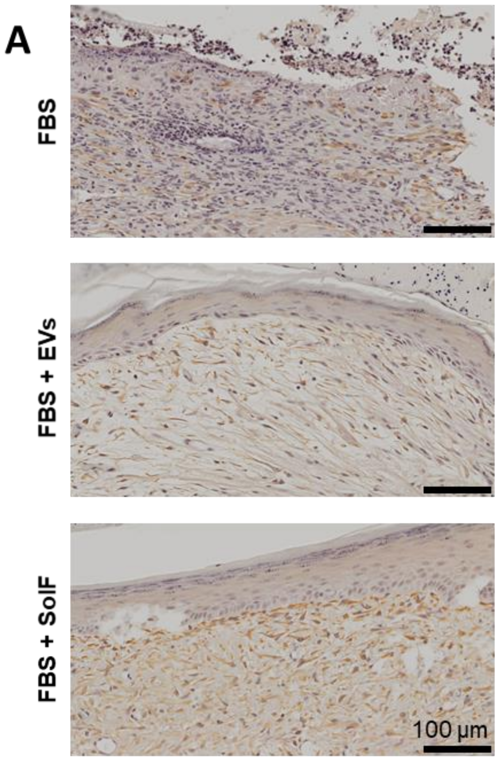

B

Transplant group	FBS	FBS + EVs	FBS + SolF
**human origin**	3/4	4/4	3/4
**cell organization**	1/4	4/4	1/4
**epidermal thickness [µm]**	141.2 ± 33.3	110.72 ± 29.5	129.73 ± 23.6
**vessels / 0.1mm²**	2.3 ± 0.38	5.2 ± 0.58	3.6 ± 0.32
**dermal score**	2.0 ± 1.2	5.0 ± 1.5	6.2 ± 1.7

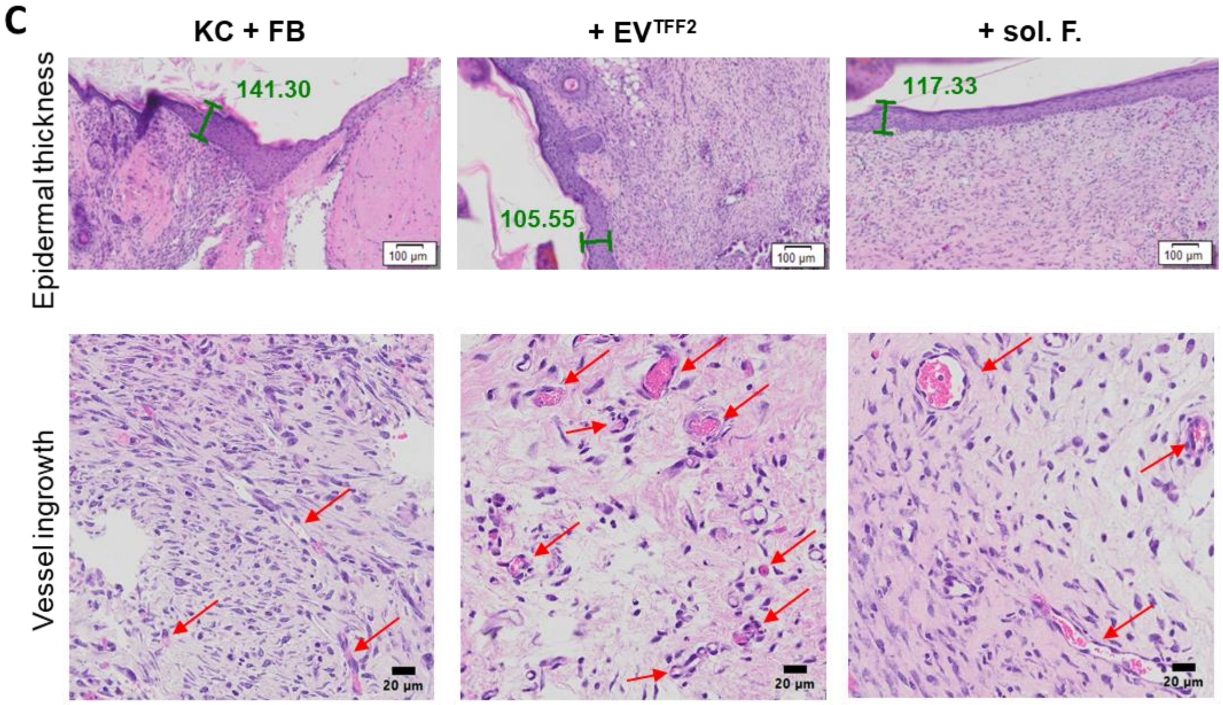

Figure S8: PLX-EVs promote skin regeneration in vivo. (**A**) Histology of human skin cell grafts on mice stained with anti-human vimentin antibody (green) and anti-human CD44 antibody (red) to verify human origin of the graft; n = 4 animals per group. Scale bar 200 µm. (**B**) Overview of important parameters to determine skin quality: human transplant establishment, proper organization of the different skin layers determined by scoring, epidermal thickness, vessel density/0.1mm² and overall dermal score[33]. (**C**) Representative images for human epidermal thickness and murine vessel ingrowth quantification in skin transplants. Thickness of epidermis was measured at the indicated example position (green lines) and the thickness in µm is stated. One example image of areas for each group used for vessel counting is shown as indicated. Only closed lumen surrounded by endothelial lining and filled with erythrocytes were counted as vessels (indicated by red arrows).

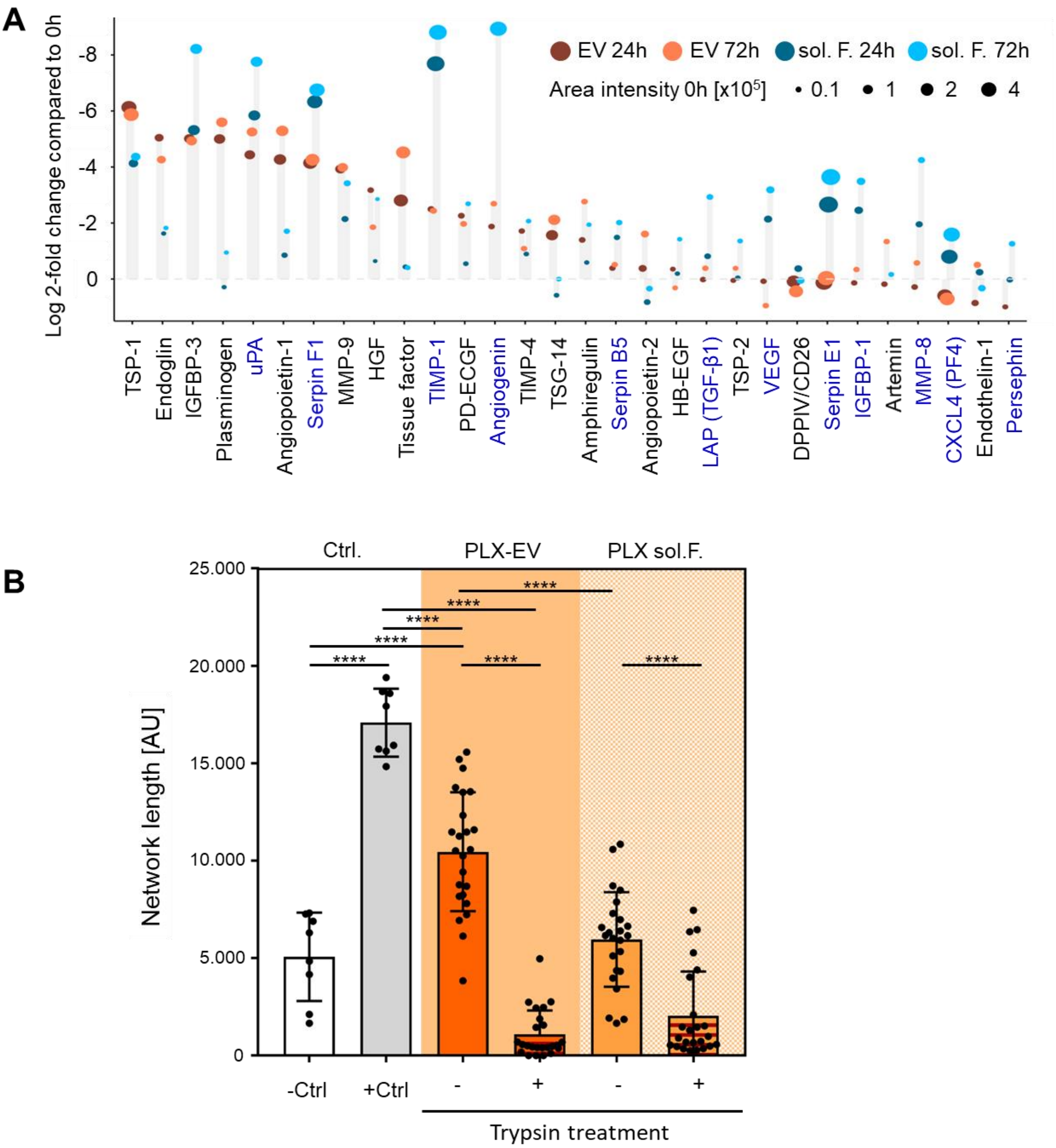

Figure S9: Corona functions are sensitive to protease digestion. (**A**) Angio-proteome profiling of EVs (reddish dots) vs. sol. F. (bluish dots) after protease treatment, compared to untreated control samples. Dots indicate Log-2-fold protein level change in equal sample volumes (e.g., Log-8 representing 256-fold less). Color code as indicated. Dot area intensity illustrates protein profiler spot area multiplied by the mean spot intensity of the untreated sample. T-test followed by Benjamini/Hochberg correction comparing EV^{TFF2} vs. sol. F. preparations after 24 and 72 hours in the absence or presence of 1 mg trypsin/mg protein, respectively. Adjusted p value < 0.05; blue-colored proteins indicating significant protection from degradation in the presence of EVs. (**B**) Endothelial network formation induced by PLX EVs or soluble factors with and without protease treatment for 30 minutes. ANOVA with Sidak correction for multiple comparisons; n = 3, two replicates, quadruplicate wells; **** p < 0.0001.

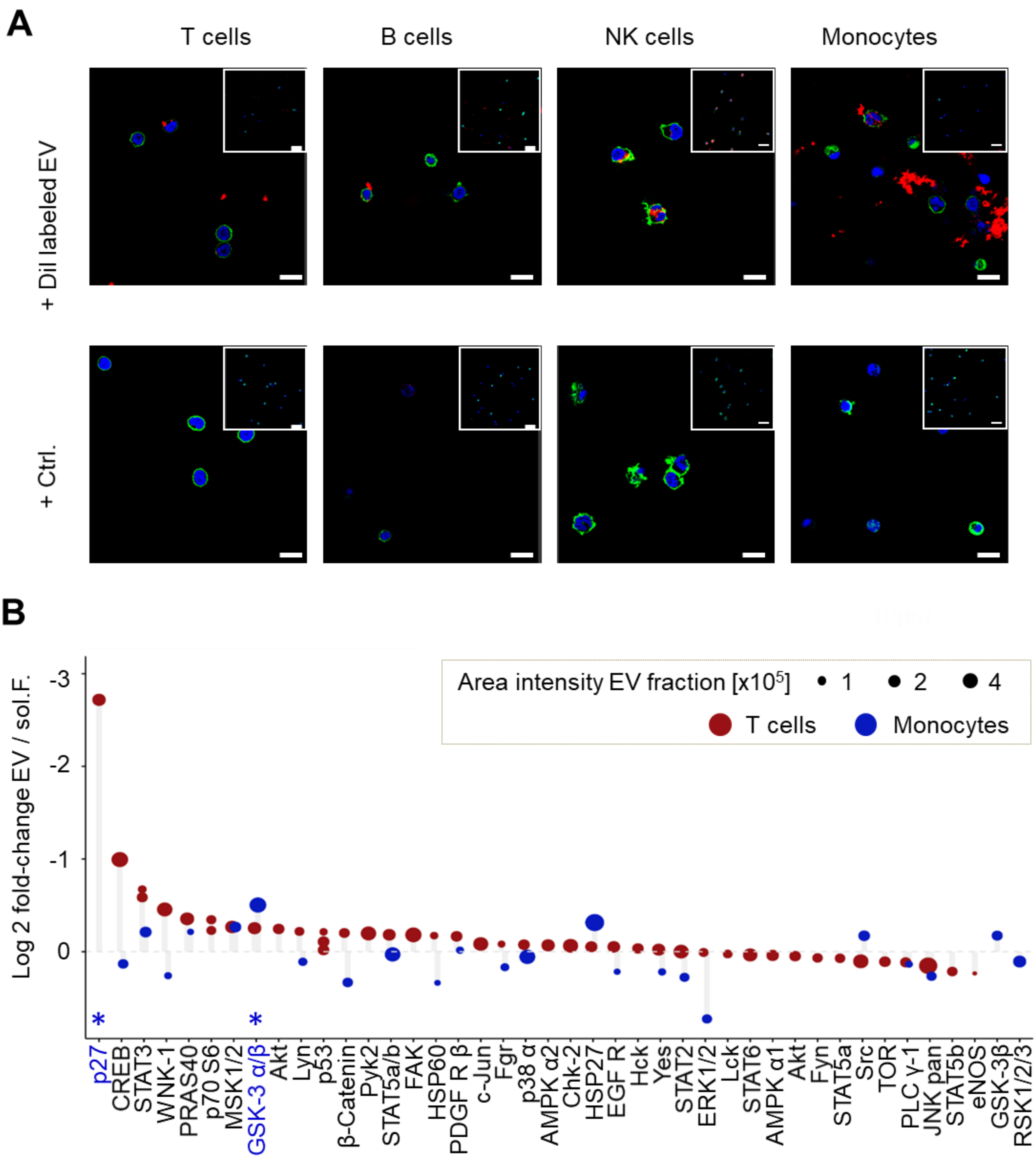

Figure S10: Immunomodulation and cell signaling by EVs: (**A**) Representative overview area after incubation of sorted T cells, B cells, NK cells and monocytes, stained with phalloidine (green) and DAPI (blue) and incubated for 24 hours with DiI (red) labeled PLX EVs or control (Ctrl.) is shown. Scale bar 10 µm. Insert represents overview with 40 µm scale-bar. (**B**) Sorted T cells and monocytes were treated with protein-TFF2 EVs for 15 minutes before cell lysis and kinome profiling as described in methods (n = 3). Significant changes for T cells are highlighted in blue text (p27 and GSK-3 α/β) as analyzed by pairwise T tests (*$p < 0.05$).

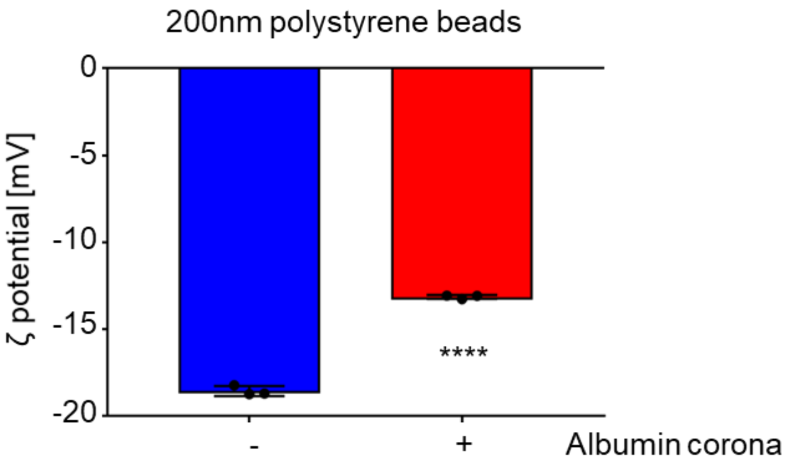

Figure S11: Zeta potential of polystyrene beads. Zeta potential of 200 nm sized polystyrene beads with (+) and without (-) protein corona (human albumin, 2 mg/mL) measured in triplicates by TRPS. Mode of distribution used for statistical analysis using t-test (n = 3; ****p < 0.0001).

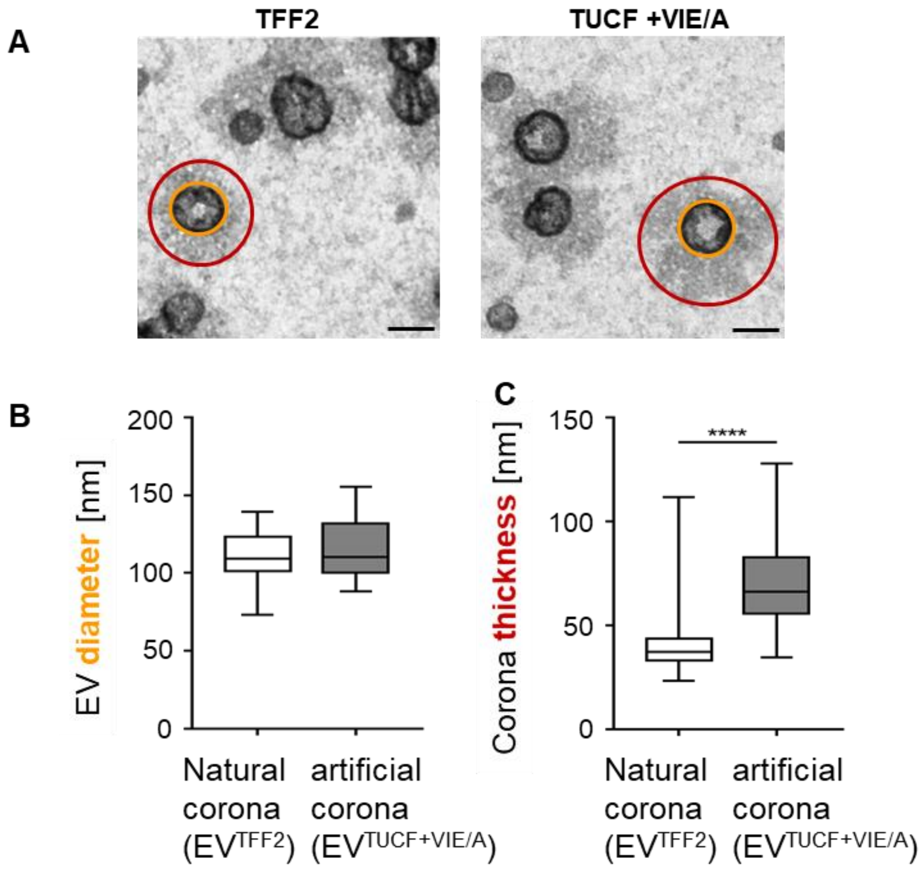

Figure S12: Quantification of EV corona thickness. (**A**) Representative images for corona quantification in negative-contrast electron microscopy images. Yellow circles indicate EV margins and red circles the corona dimension. (**B**) Diameter of EVs measured from one overview picture per donor from three individual donors. The individual data points are shown in Fig.7B. (**C**) Corona thickness calculated as the difference between the radius of the corona minus the radius of the EV. Displayed as box whisker blots with minimum to maximum whiskers. We analyzed n = 36 EV^{TFF2} and n = 56 EV^{TUCF} + VIE/A. Statistics were calculated using nonparametric Mann-Whitney test for unpaired samples, ****p < 0.0001.

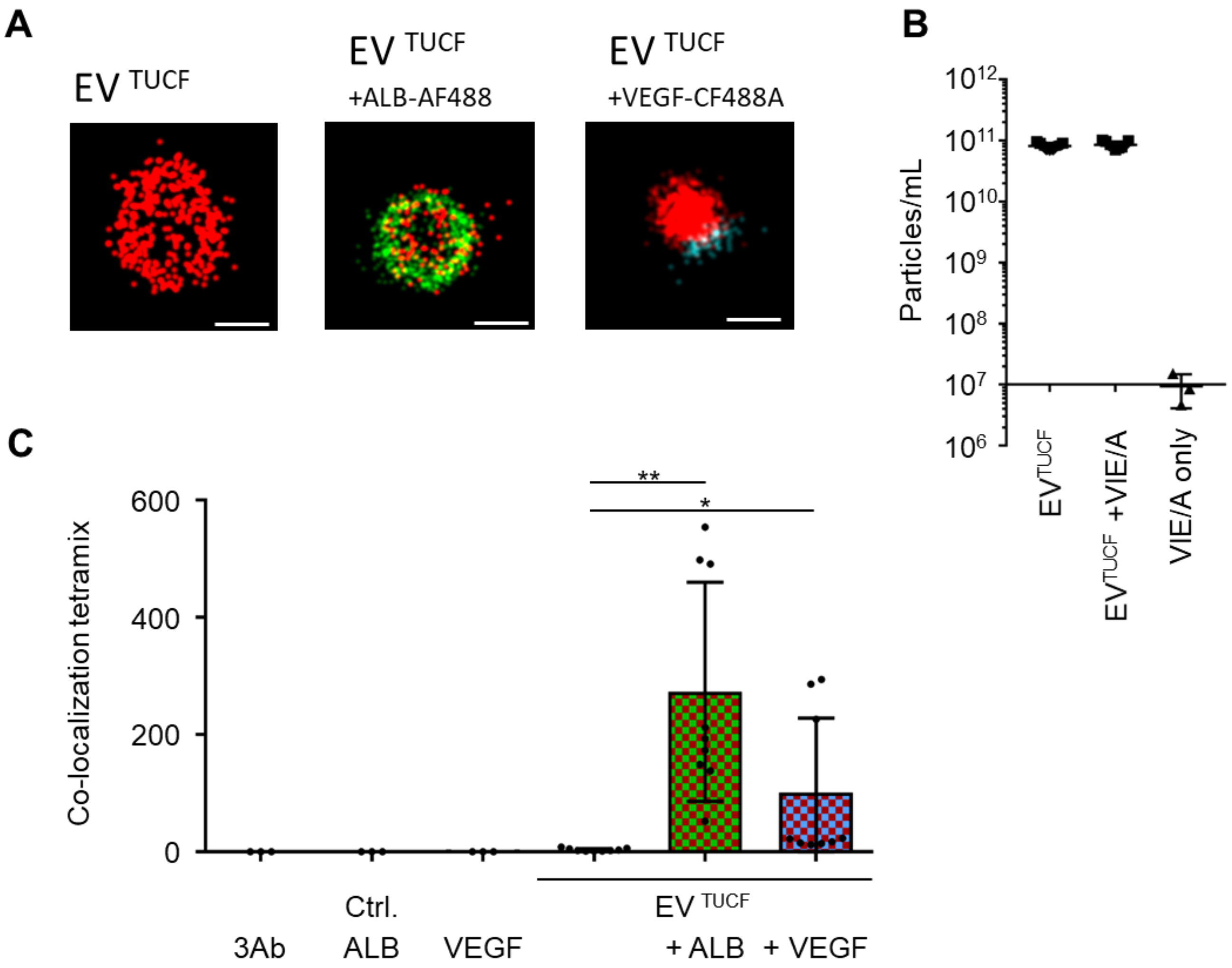

Figure S13: Corona visualization and particle counts during EV corona formation. (**A**) Example pictures of albumin (ALB)-AF488 (green) and VEGF-CF488A (cy-flour, pseudo-color turquoise) protein corona at EVs stained against major tetraspanins CD9-AF647, CD63-AF647 and CD81-AF647 (tetramix) as indicated. Scale bars 100 nm. (**B**) Particle count for EV^{TUCF} before and after corona reconstitution with VIE/A measured by tunable resistive pulse sensing (TRPS) for three donors in triplicates. X-axis intersects y-axis at limit of quantification (LOQ; mean background + [9x standard deviation of background]). (**C**) Quantification of co-localizations between tetraspanin positive clusters (EVs) and ALB-AF488 and VEGF-CF488 signal. Three images from three individual PLX donors were quantified using CODI software. Statistical analysis was performed using paired t test (**$p < 0.01$).

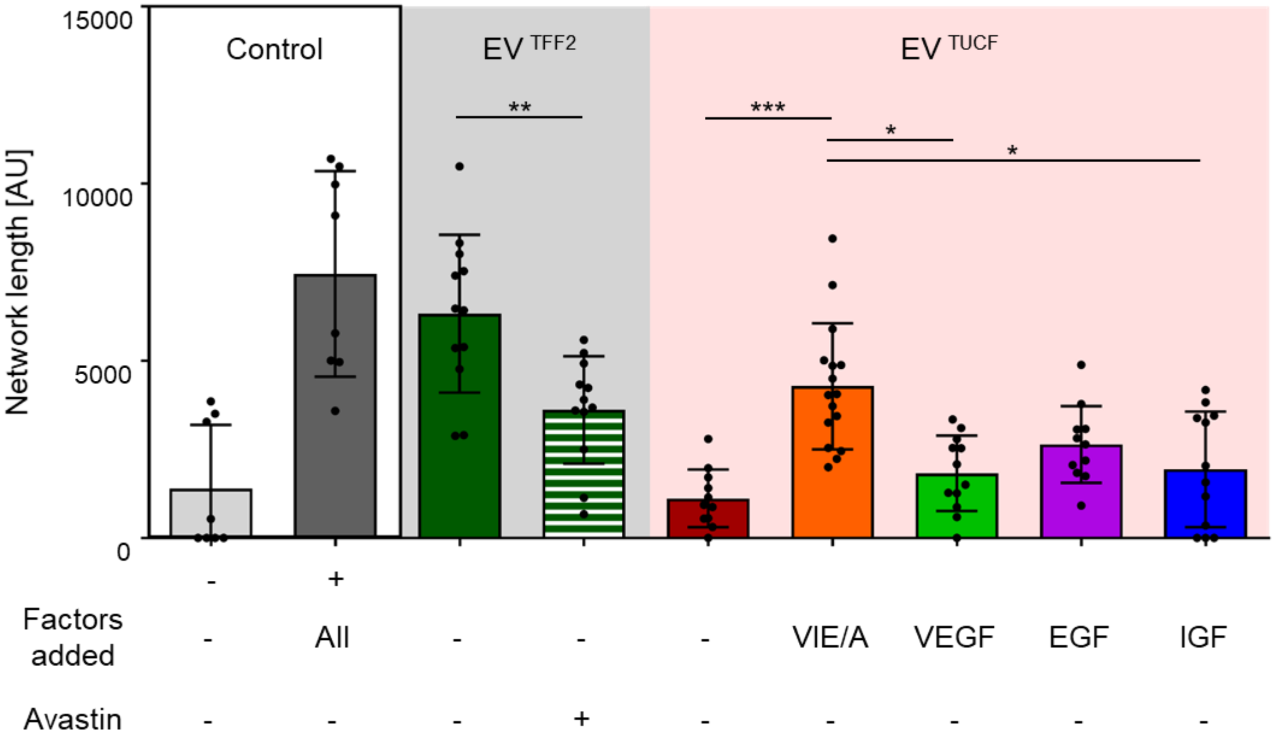

Figure S14: Pro-angiogenic function of PLX EVs depends on different corona components. Endothelial cell network formation was compared to control conditions (left two columns) in the absence (-) or presence (+) of commercial 'single quots' containing pro-angiogenic factors, including VEGF, in optimized concentration. Treatment of endothelial cells with EV^{TFF2} (green bars) can fully replace optimized pro-angiogenic factors. Anti-VEGF antibody (Avastin; green hatched bar) partly but significantly inhibited EV function. Protein corona of EV^{TUCF} (red bar) was re-established with single factors or a mix of them in Albumin (VIE/A) as indicated. Data for EV preparations from three individual donors in four replicates were analyzed. One-way ANOVA with Tukey correction for multiple comparisons (*p < 0.0332, **p < 0.0021, ***p < 0.0002).

WESTERN BLOT SUPPLEMENT

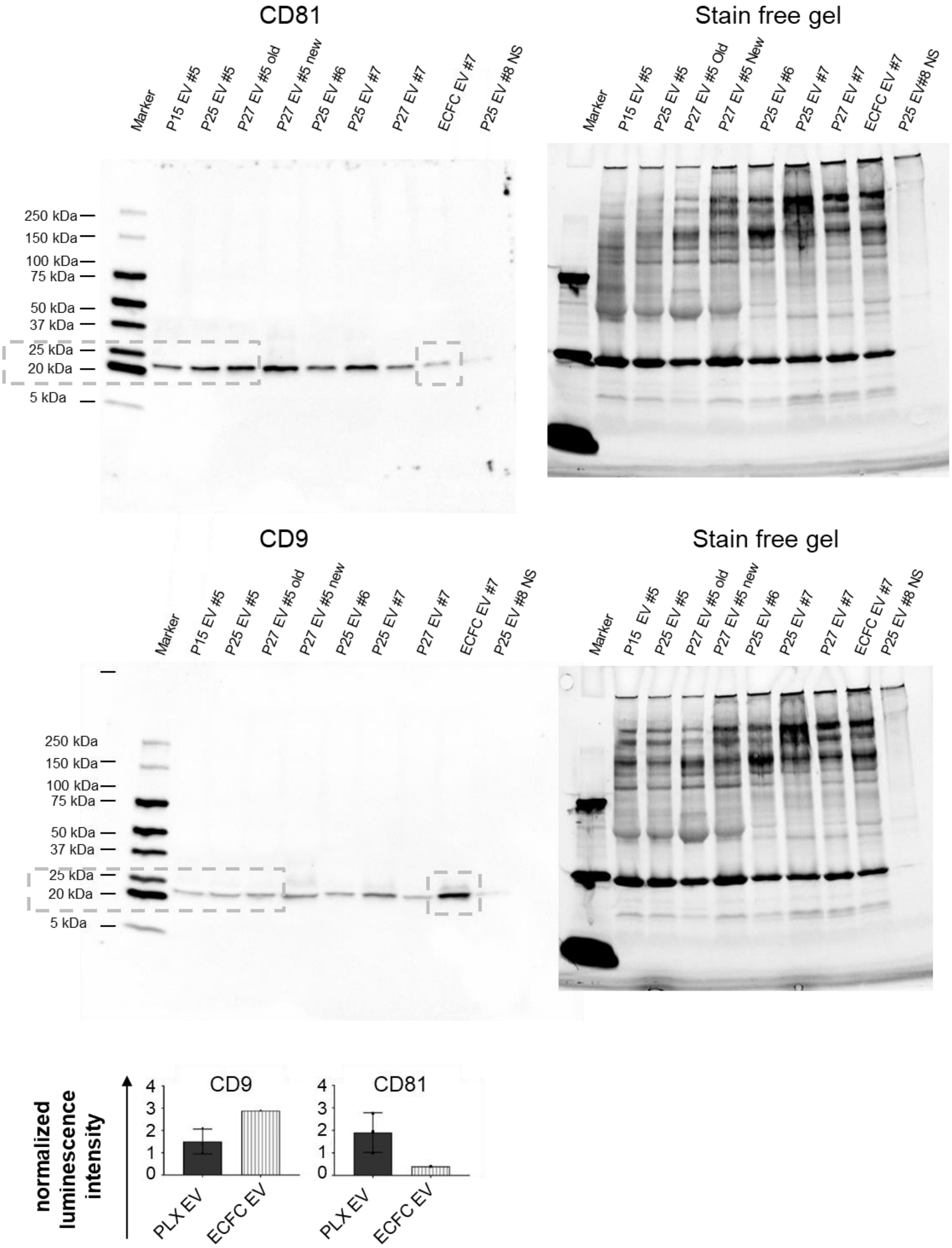

Supplementary Western blot data A: EV identity and purity determined by western blot: Whole membrane and gel of CD81 and CD9 immunoblots in Figure 3A.

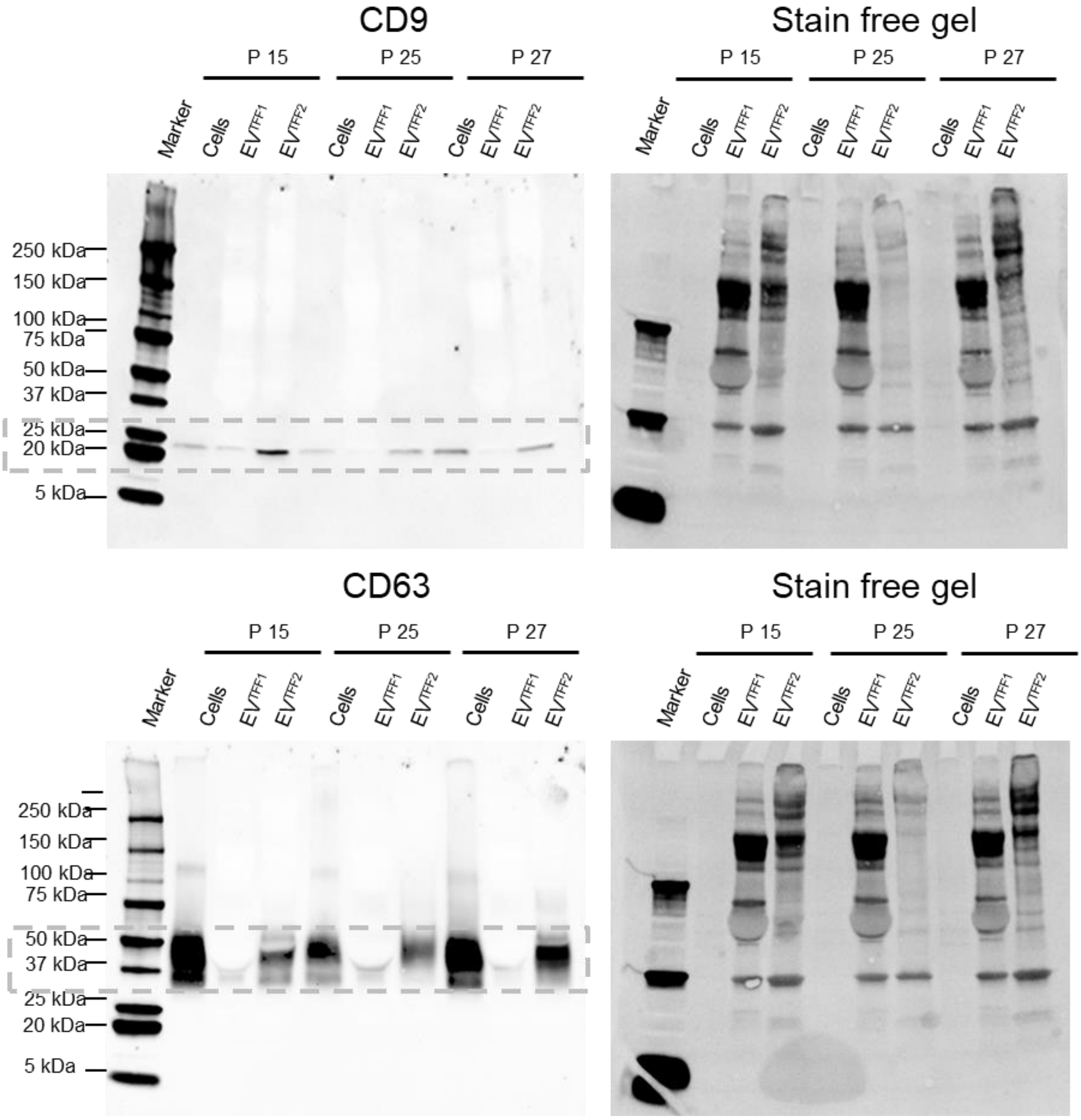

Supplementary Western bot data B: EV identity and purity determined by western blot: Whole membrane and gel of CD9 and CD63 immunoblots in Figure 3B.

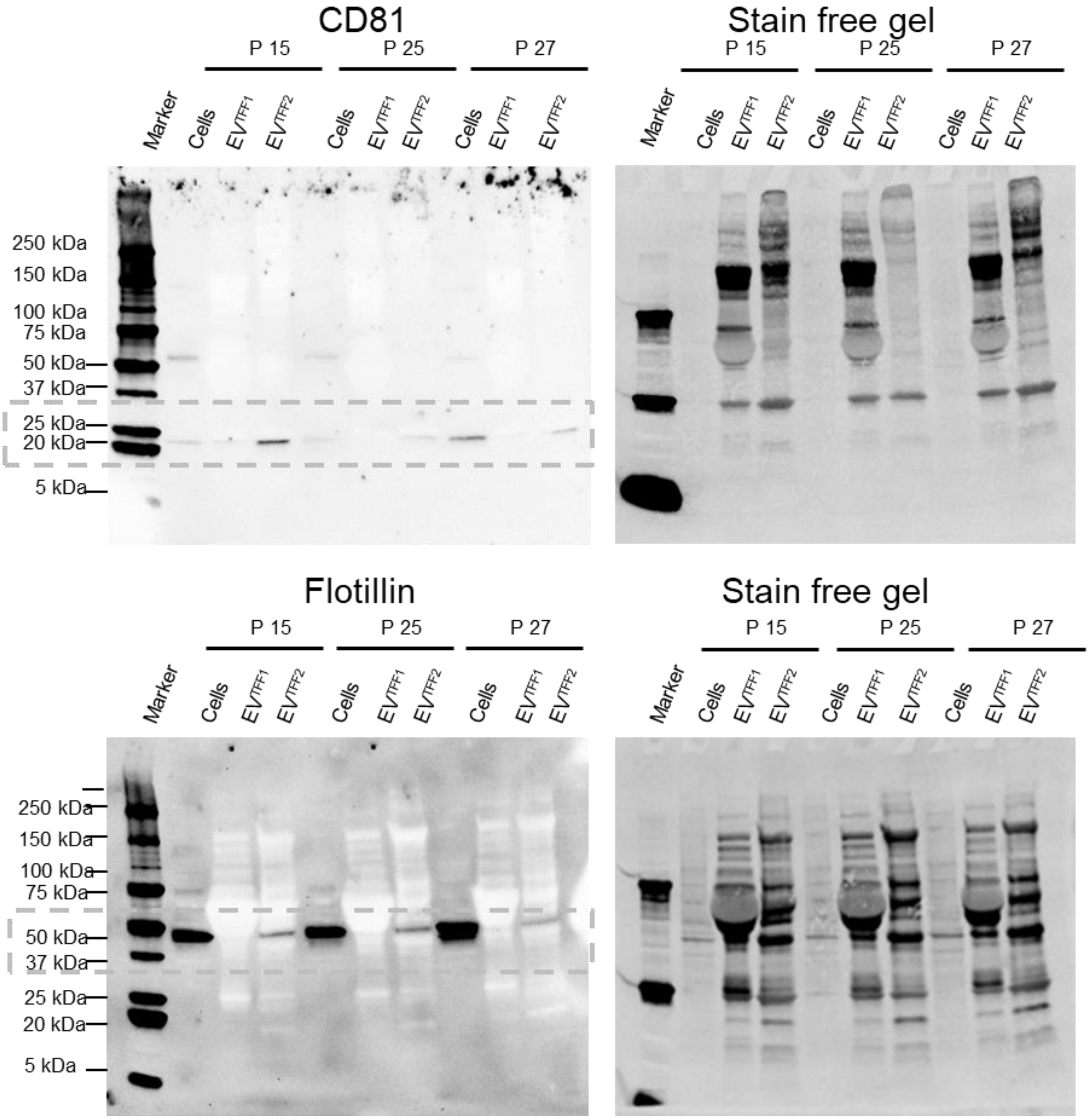

Supplementary Western bot data C: EV identity and purity determined by western blot: Whole membrane and gel of CD81 and flotillin immunoblots in Figure 3B.

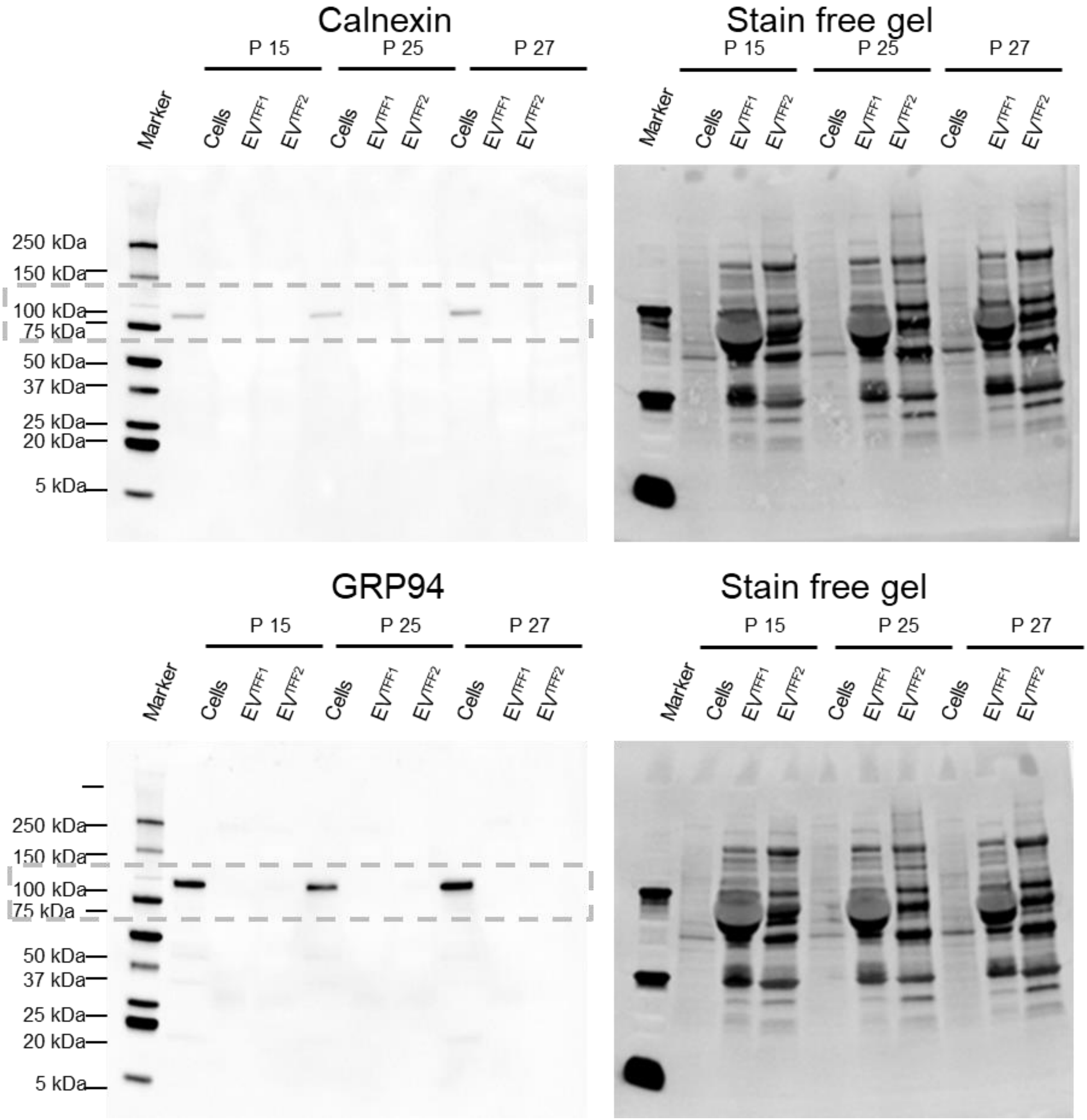

Supplementary Western bot data D: EV identity and purity determined by western blot: Whole membrane and gel of calnexin and GRP94 immunoblots in Figure 3B.

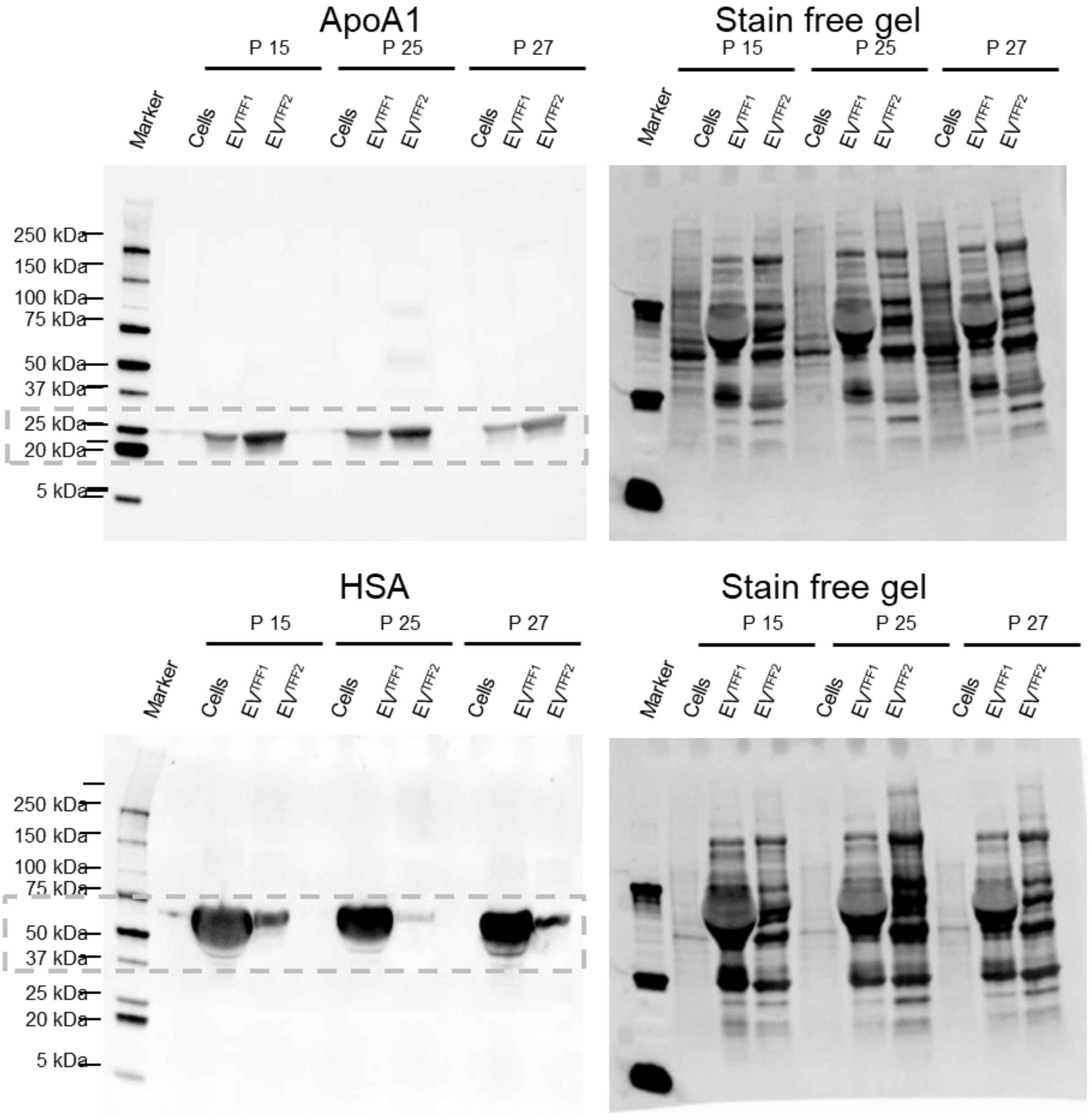

Supplementary Western bot data E: EV identity and purity determined by western blot: Whole membrane and gel of ApoA1 and HSA immunoblots in Figure 3B.

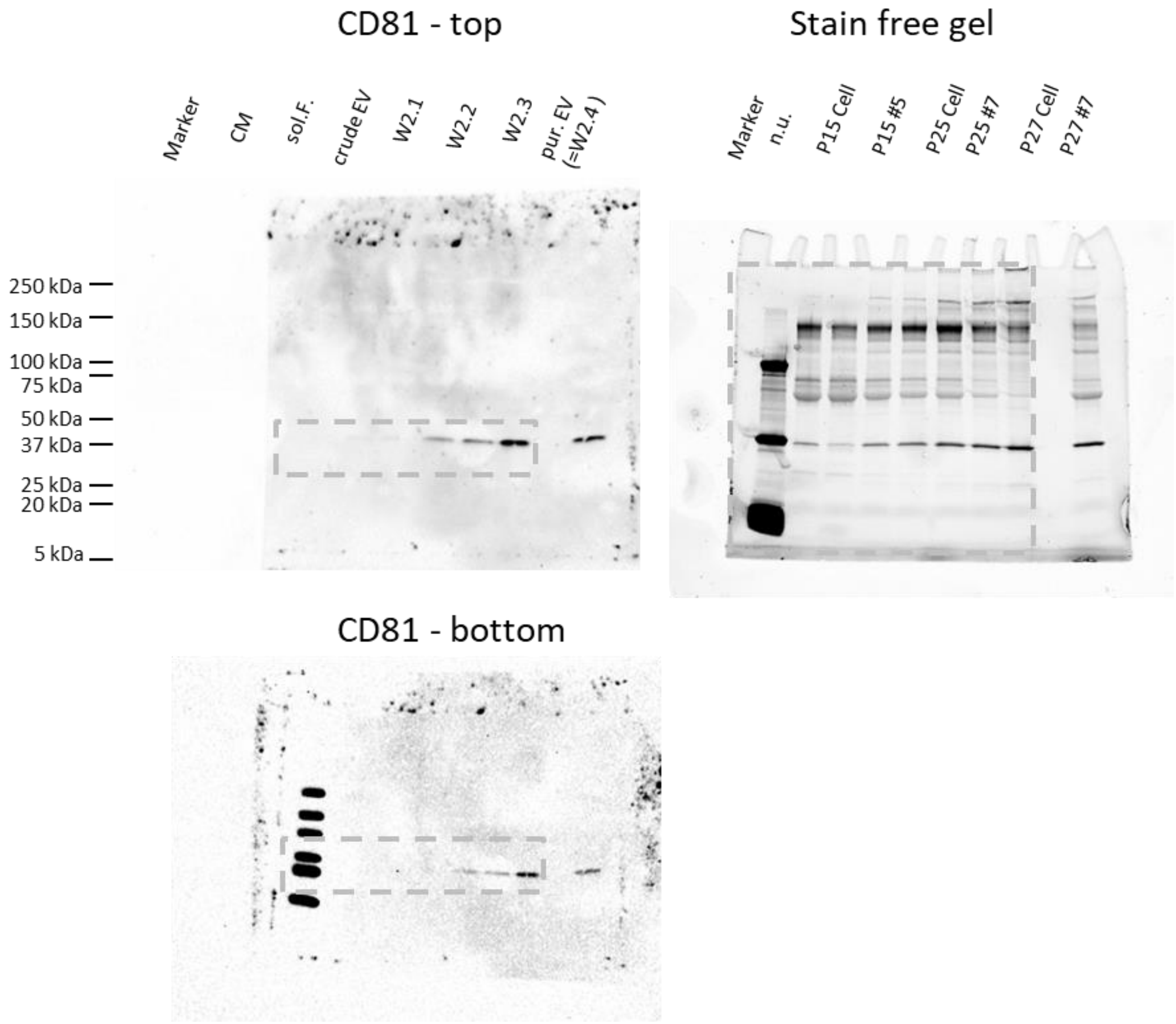

Supplementary Western bot data F: EV enrichment determined by western blot: Whole membrane and gels of CD81 immunoblots in Figure 3C.

Table S1: Antibodies and dilutions used in western blots.

Name	Supplier	Clone	Isotype	Concentration	WB dilution
**Apolipoprotein A1**	GeneTex	polyclonal	IgG	0.66 mg/mL	1:1320
**Calnexin**	Cell Signaling	C5C9	IgG	unknown	1:1000
**CD9**	Invitrogen	MM2/57IVA50	IgG2	1 mg/mL	1:50
**CD63**	Thermo Fisher	TS63	IgG1	0.5 mg/mL	1:1000
**CD81**	Bio-Rad	1D6	IgG1	1 mg/mL	1:500
**Flotillin 1**	BD	Flotillin-1	IgG1	0.25 mg/mL	1:1000
**GRP94**	Bio-Rad	polyclonal	IgG	unknown	1:2000
**Human Serum Albumin**	Thermo Fisher	KT11	IgG1	1 mg/mL	1:1000

Table S2: Antibodies and dilutions used for flow cytometry.

Antibody/ dye	Flourochrome	Clone	Isotype	Concentration	Species	Clonality	Supplier
**7AAD**							eBioscience
**CD3**	eFluor450	SK7	IgG1, k	100 µg/mL	mouse	monoclonal	eBioscience
**CD14**	APC-H7	MΦP9	IgG2b, k	25 µg/mL	mouse	monoclonal	BD
**CD19**	APC	HIB19	IgG1	6,25 µg/mL	mouse	monoclonal	eBioscience
**CD45**	Krome Orange	J.33	IgG1	100 µg/mL	mouse	monoclonal	Coulter
**CD56**	PE-Cy7	CMSSB	IgG1, k	25µg/mL	mouse	monoclonal	eBioscience

Table S3: Sample details for proteomic analysis: Protein content (Bradford) and particle count as measured by TRPS for samples used for proteomic analysis.

Sample	Protein [mg/mL]	EV count [particles/mL]	Protein IDs [count] unlabelled - FDR 1%
α-MEM*/TFF	1.5	1.18×10^{8}	631
P15 sol.F.	1.1	n.a.	405
P15 EVs^{TFF1}	15.3	6.45×10^{10}	549
P15 EVs^{TFF2}	2.6	8.68×10^{10}	1,095
P25 sol.F.	1.5	n.a.	559
P25 EVs^{TFF1}	11.7	7.45×10^{10}	401
P25 EVs^{TFF2}	2.4	2.46×10^{11}	1,168
P27 sol.F.	1.3	n.a.	448
P27 Evs^{TFF1}	38.5	8.17×10^{10}	814
P27 EVs^{TFF2}	2.1	1.58×10^{11}	622

Table S4: Quantitative proteomic characterization of PLX secretome fractions.

Go terms used for labelling in Volcano Plot			
**Movement**	**Immune modulation**	**Angiogenesis**	**Extracellular vesicles**
cell migration GO:0016477	regulation of inflammatory response GO:0050727	positive regulation of angiogenesis GO:0045766	endocytic vesicle lumen GO:0071682
morphogenesis of a polarized epithelium GO:0001738	regulation of immune effector process GO:0002697	sprouting angiogenesis GO:0002040	vesicle GO:0031982
actin filament organization GO:0007015	neutrophil mediated immunity GO:0002446	regulation of angiogenesis GO:0045766	transport vesicle GO:0030133
postsynaptic actin cytoskeleton organization GO:0098974	leukocyte migration GO:0050900	cell-substrate adhesion GO:0031589	vesicle-mediated transport GO:0016192
skeletal muscle contraction GO:0003009	humoral immune response GO:0006959	extracellular matrix organization GO:0030198	vesicle docking involved in exocytosis GO:0006904
microtubule-based process GO:0007017	antigen processing and presentation GO:0019882	positive regulation of angiogenesis GO:0045766	vesicle transport along microtubule GO:0047496
cell chemotaxis GO:0060326	complement activation GO:0006956	negative regulation of angiogenesis GO:0016525	vesicle fusion GO:0006906
	positive regulation of B cell activation GO:0050871	negative regulation of cell migration involved in sprouting angiogenesis GO:0090051	vesicle docking GO:0048278
		negative regulation of sprouting angiogenesis GO:1903671	vesicle docking involved in exocytosis GO:0006904
			exocytic vesicle GO:0070382
			transport vesicle membrane GO:0030658
			clathrin-coated vesicle membrane GO:0030665
			extracellular vesicle GO:1903561
